# TGF-β-producing tBregs are associated with poor survival rates in PTGS2-high cancers

**DOI:** 10.1038/s41419-026-09045-w

**Published:** 2026-06-26

**Authors:** Kavitha Premkumar, Soundharya Ramu, Mohit Kumar Jolly, Bhavani S. Shankar

**Affiliations:** 1 Immunology Section, Radiation Biology & Health Sciences Division, Bio-Science Group, Bhabha Atomic Research Centre, Mumbai, India; 2 Interdisciplinary Mathematics Initiative, Indian Institute of Science, Bengaluru, India; 3 Department of Bioengineering, Indian Institute of Science, Bengaluru, India; 4 Homi Bhabha National Institute, Mumbai, India

**Keywords:** Tumour immunology, Cancer microenvironment

## Abstract

The cues that cause B cells to adopt a regulatory phenotype are unknown. We previously reported on the regulatory B-T axis in mouse fibrosarcoma. This work demonstrates the presence of TGF-β-generating Bregs in the sarcoma microenvironment as well as variable gene expression depending on TGF-β levels. Breg depletion reduced tumor-evoked Bregs and progression of WEHI-164 fibrosarcoma, which was reversed by adoptive transfer of B cells. Tumor-derived PGE2 acting via EP4/STAT3 signaling is a key driver of tBreg formation, ultimately resulting in increased Tregs. Only the TGF-β^+^ tBregs caused T cell suppression. PGE2 activated STAT3 in a non-canonical manner since pre-treatment of B cells with JNK and NF-κB inhibitors completely restored tBreg-induced T cell suppression. The COX-2 inhibitor NS-398 prevented the generation of tumor-evoked Bregs, resulting in a lower tumor burden, fewer Tregs, and improved T cell responses. TGF-β-producing Bregs were identified in sarcomas and other cancers with elevated PGE2. The B cells from PTGS2^hi^ cancers displayed unique Breg signatures when compared to PTGS2^lo^ cancers. TCGA analysis showed that the Breg signature was higher in patients with high PTGS2 levels, which was also associated with a poor survival rate. In summary, we found that the tumor-derived PGE2 activates non-canonical MAPK/STAT3 in B cells, leading to the formation of TGF-β-generating Breg cells. PTGS2^hi^ Breg^hi^ patients were associated with a poor survival rate, while the COX-2 inhibitor NS-398 prevented tBreg generation and reduced tumor burden. This could lead to the development of novel strategies for reducing tBreg production in cancer.

## Introduction

Soft tissue sarcomas are tumors of mesenchymal origin that are heterogeneous in terms of anatomical sites, histology, and molecular features, with multiple subtypes, and can impact individuals across all age groups [1, 2]. Up to 30% of the STS patients who received multimodal therapies later develop a hard-to-treat metastatic condition. When it comes to immunotherapy, only a few types of STS have shown major responses, but the main barriers are heterogeneity and a lack of complete understanding of its immune microenvironment [3]. The Cancer Genome Atlas Research Network analyzed 28 immune signatures across 206 adult STSs and identified positive prognoses associated with NK cell, CD8^+^ T cell, and dendritic cell scores in 6 different types of sarcoma [4]. Petitprez et al. identified five distinct immune phenotypes in a study on 608 STSs, with A and B being immune-low, D and E as immune-high, and C as the highly vascularized group [5]. The authors conclude that B-cell infiltration with the formation of tertiary lymphoid structures (TLS) was associated with improved survival. However, correlation of immune histochemical assessment of CD20- and CD19-positive cells with prognostic association has yielded both negative and positive correlations with survival [6]. In addition, several signaling pathways, like lactate and TGF-β, inhibit the formation of tertiary lymphoid structures [7, 8]. All of these results suggest that B cells in sarcomas are influenced by the soluble mediators of the tumor microenvironment and need further investigation.

B cells are a major component of the immune system known to produce antibodies, secrete immunoregulatory cytokines, and serve as antigen-presenting cells [9]. Besides these functions, a subset of B cells, called regulatory B cells (Breg), have immunosuppressive properties [10]. Even though these Bregs are well investigated in the etiology of autoimmune diseases and infections, emerging data points to their participation in cancer [11–13]. Bregs in the tumor have been shown to have pro-tumor effects by inhibiting T cells and NK cells and enhancing the activity of Tregs and other immunosuppressive cells [14, 15]. Anti-CD20-mediated B cell depletion decreased tumor burden associated with increased Tregs in a methylcholanthrene-induced murine fibrosarcoma model [15]. The existence of similar immunosuppressive B cells is reported in a limited number of preclinical cancer models [16, 17]. A pro-tumoral relationship of B cells in human sarcoma has been reported by Smolle et al., who showed a negative correlation of CD20^+^ B cells with local recurrence and no influence on overall survival based on studies using 1266 tissue core samples from 188 STS patients [18]. Such pro and anti-tumoral effects of B cells in the STS microenvironment indicate the complexity and heterogeneity of B cell populations. These data also highlight the importance of the spatial distribution of B cells in TME, which could determine the outcome of the immune response rather than the mere presence of these cells. Hence, it is very important to identify the mediators in the tumor microenvironment that influence the B cells of the TME to acquire such varied roles.

Recently, we have reported that WEHI-164 fibrosarcoma induced CD19^+^CD81^+^CD25^hi^PD-L1^hi^CD45R^hi^ CD85^hi^ MHCII^hi^CD27^hi^ and P-STAT3^+^ Breg cells that secrete IL-10 and TGF-β. These tumor-evoked Bregs (tBregs) suppressed T cell responses and converted CD4^+^ cells into Treg cells through TGF-β-mediated signaling [19]. In this study, we demonstrate that TGF-β-producing tBregs in sarcoma have enhanced expression of immunosuppressive markers when compared to low TGF-β-expressing B cells. Despite the fact that several studies have found Bregs in cancers, the factors responsible for their generation are not known. Bodogai et al. demonstrated that 5-lipooxygenase metabolites induced tBregs in 4T1 mammary cancer [20]. Other investigators have found that cytokine IL-18, fatty acids, or autophagosomes generated Bregs in different cancers [17, 21]. In this study, we show that the WEHI-164 tumor cells secrete prostaglandins (PG), which induce TGF-β1-producing tBreg cells in BALB/c mice. Both in vitro and in vivo, tBreg induction and immunosuppression were prevented by pharmacologically blocking EP receptor signaling in B cells or inhibiting COX2 in tumor cells. In addition, sarcomas and other cancers with elevated PGE2 had a higher proportion of TGF-β-producing Bregs displaying unique Breg signatures. The proportion of Breg was higher in patients with high PTGS2 levels, which was associated with a poor survival rate. Though PTGER4 is known as a negative regulator of BCR signaling, and elevated levels of PGE2 have been known to sensitize B lymphocytes to antigen-specific tolerogenic signals, how this suppressive effect of PGE2 on B cells will affect anti-tumor immunity and cancer progression was not clear [22, 23]. Through this study, we report the pivotal role of COX2 and tumor-derived PGE2 in inducing immunosuppressive tBreg and Treg cells, leading to increased cancer progression. To the best of our knowledge, this is the first report of PGE2-induced tBregs in cancer.

## Results

### Breg cells expressing TGFB1 in sarcoma

We previously demonstrated that WEHI-164 fibrosarcoma cells induced the generation of CD19^+^CD81^hi^CD86^hi^CD25^+^CD45R^+^STAT3^+^IL-10^+^TGF-β^+^ tumor-evoked Bregs (tBregs) [19]. These tBregs suppressed T cell proliferation and induced Tregs in a TGF-β-dependent manner. SB431542, a pharmacological inhibitor of the TGF-β receptor, restored T cell responses and reduced tumor burden by abrogating the immunosuppressive function of tBregs [19]. However, the existence of such regulatory B cells in sarcomas and other cancers and the specific mechanism of their induction are not well documented.

**Fig. 1:**
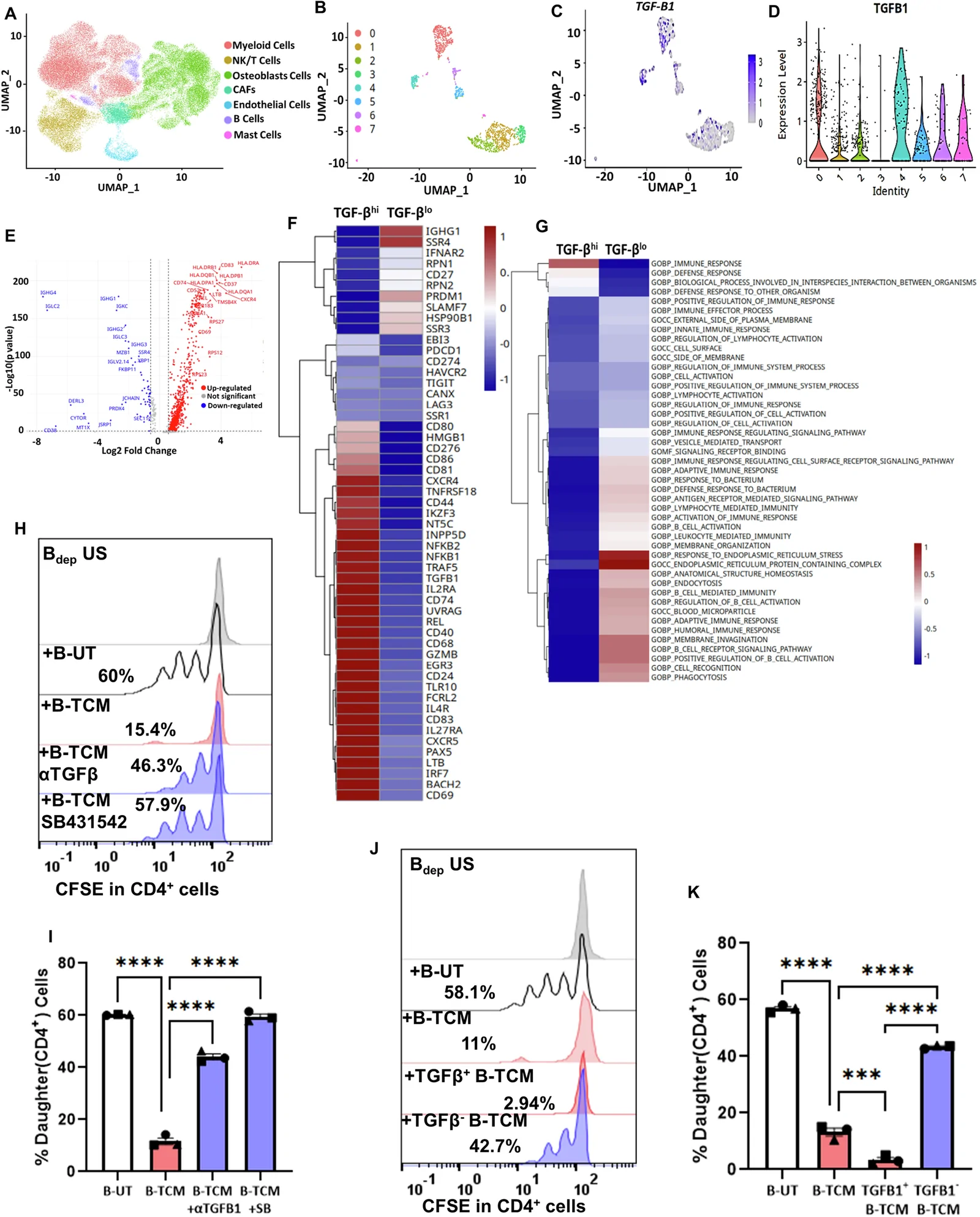
TGF-β-expressing Breg cells in sarcoma. **A** UMAP projection of cell clustering information, color-coded by clusters. **B** UMAP plot representation of the B-cell subpopulation, colored and labeled by UMAP sub-cluster annotations. **C** UMAP representation and **D** feature plot visualizing the expression of the *TGFB1* gene in the B cell subset. **E** Volcano plot showing DEGs in TGF-β^hi^ and TGF-β^lo^ B cells. **F** Heatmap presenting the expression of the top 20 upregulated and downregulated genes in the TGF-β^hi^ B cells. **G** Heatmap depicting the normalized expression scores of the significantly upregulated and downregulated pathways in the TGF-β^hi^ B cells. T cell response in the presence of tBregs generated from WEHI-164 fibrosarcoma. B cells were cultured with 20% TCM (B-TCM) for 72 h and then co-cultured with CFSE-labeled, stimulated B_dep_ cells along with neutralising anti-TGFβ1 antibody or TGFβRI inhibitor SB431542. **H** Representative flow histograms of CFSE dye dilution in CD4^+^ gated T cells from different treatment groups. **I** Bar diagram of T cell proliferation (*n* = 3). B- TCM cells were sorted into TGFβ^+^ and TGFβ^-^ B cells using MACS and co-cultured with CFSE-labeled, stimulated B_dep_ cells. **J** Representative flow histograms of CFSE dye dilution in CD4^+^ gated T cells from different treatment groups. **K** Bar diagram of T cell proliferation (*n* = 3).

In order to identify the presence of TGF-β^+^ Bregs in human sarcoma, we downloaded publicly available single-cell RNA sequencing data from 6 osteosarcoma (OS) patients (GSE162454). After quality control for all the samples, the cell annotation showed different immune cell subpopulations, including B cells (Figs. 1A and S1A, supporting information). B cells were associated with clusters 13 and 14 expressing *MS4A1* and *CD79A* (Fig. S1A, supporting information). These two B cell clusters were re-clustered into seven subclusters (Fig. 1B). In these subclusters, we looked for the expression of *TGFB1*. Most of the clusters expressed *TGFB1* (Fig. 1C), and among them, the clusters 0 and 4 expressed the highest level and thereby corresponded to TGF-β^hi^ B cells (Fig. 1D). We observed that the other clusters can be split into TGF-β^mid^ (clusters 5, 6, and 7) and TGF-β^lo^ (clusters 1, 2, and 3) B cells (Fig. 1D). Differentially expressed genes (DEGs) were analyzed between TGF-β^hi^ and TGF-β^lo^ B cells to identify genes specific to TGF-β^hi^ B cells. We observed 853 DEGs between these two groups, out of which 632 were upregulated (log2(FC) > 0.25, FDR < 0.05) and 221 were downregulated (log2(FC) < -0.25, FDR < 0.05). The volcano plot and heatmap show DEGs that are upregulated or downregulated to 1.5-fold or above (log2(FC) ≥ 0.585 or log2(FC) ≤ –0.585) (Figs. 1E and S1B, supporting information).

TGF-β^hi^ B cells had high levels of *CD19* expression with down-regulation of *CD38*, *JCHAIN*, and *SDC1* and absence of *CD27*, indicating that they are naive but not plasma cells. These cells also expressed high levels of immature B cell markers *TCL1A*, *IGHD*, *SELL*, and *FCMR*. Expression of *CD69* and *CD83,* along with increased chromatin remodeling genes (*CHD1*, *AIRD1A*, *BAF*, *HDAC1*, *HDAC2*, and *JMJDC1*) and ribosome biogenesis genes (*MYC*, *NPM1*, *NC1*, *NOP53*, *DDX17*, *DDX21*, *RPS* genes, and *RPL* genes), is a hallmark of cell growth and differentiation and indicates the activated state of TGF-β^hi^ B cells. TGF-β^hi^ B cells highly expressed MHCII- and MHCI-related genes (*HLA-DQ*, *HLA-DP*, *HLA-DR*, *HLA-A*, *HLA-B*, and *HLA-C)* and markers associated with trafficking and chemotaxis (*CXCR4*, *CXCR5*, *GPR18*, *CDC42*, and *ARPC3*), suggesting MHC-mediated antigen presentation and interaction with T cells might be one function of these cells. On the other hand, high expression of *HLA-E*, *HLA-F*, and *HLA-DO* indicates that TGF-β^hi^ B cell-T cell interaction could promote tolerogenic T cell differentiation (Fig. 1F).

DEG analysis revealed that TGF-β^hi^ B cells expressed high levels of markers associated with immature B cells, activation and proliferation, chemotaxis, and antigen presentation, along with high levels of the immunosuppressive HLA-1b class of MHCs. This suggests a possible interaction of these tolerogenic B cells (or Breg cells) with T cells. To confirm whether these cells exhibited a Breg-like phenotype, we assessed the previously reported Breg-associated markers in the DEGs of TGF-β^hi^ B cells. Most of the Breg-associated genes were highly expressed in TGF-β^hi^ B cells (Fig. 1F). One interesting factor was the upregulated NF-κB pathway genes (*NFKB1*, *NFKB2*, *REL*, *TRAF5*, and *BIRC3*). The pathway analysis shows an upregulation of metabolic pathways and biosynthetic processes and a downregulation of various B cell receptor signaling and immune response pathways in TGF-β^hi^ B cells (Figs. 1G and S1C, supporting information). Overall, these data show that TGF-β^hi^ B cells were associated with a gene expression profile related to the regulatory B cell signature.

To confirm the role of TGF-β in Breg-mediated suppression, we generated tBregs using WEHI-164 tumor-conditioned medium (B-TCM). We then inhibited TGF-β signaling in a classical Breg-T cell co-culture system using either a neutralizing anti-TGF-β antibody (1D11) (B-TCM αTGFβ) or a TGF-βRI inhibitor SB431542 (B-TCM SB431542) and assessed T cell proliferation. Both neutralizing antibody and receptor inhibitor effectively blocked TGF-β signaling in T cells and reversed tBreg-mediated suppression (Fig. 1H, I).

In addition to secreting TGF-β, the tBregs also express surface membrane-bound TGF-β in a LAP-TGFβ form (Fig. S1D, supporting information). To further establish that TGF-β^hi^ B cells are tBregs, we sorted TGF-β^+^ and TGF-β^-^ cells from tBregs using the LAP-TGFβ1 antibody, followed by MACS (Fig. S1D, supporting information). The sorted cells (TGFβ^+^ B-TCM and TGF-β^-^ B-TCM) were then co-cultured with stimulated T cells. TGF-β^+^ B-TCM cells exhibited enhanced T cell suppression as compared to unsorted B-TCM cells, whereas TGF-β^-^ B-TCM cells failed to suppress T cell proliferation (Fig. 1J, K). Collectively, these data demonstrate that TGF-β^hi^ B cells constitute the functional tBreg population responsible for immune suppression in sarcoma.

### B cell depletion in BALB/c mice reduced Treg and WEHI-164 tumor burden

**Fig. 2:**
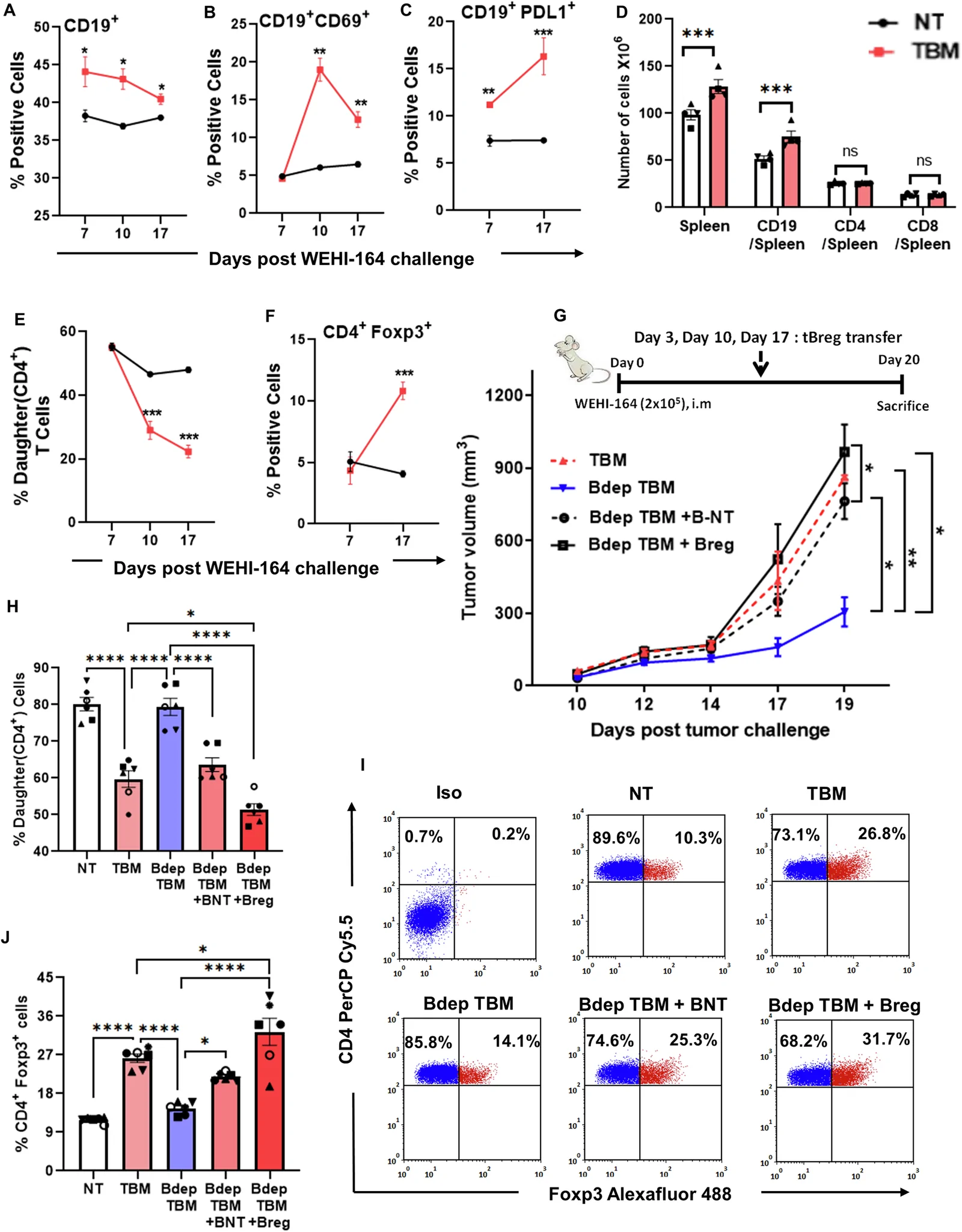
B cell depletion reduced Treg and WEHI-164 tumor burden. Splenic immune cell temporal dynamics in WEHI-164 TBM. Percentages of **A** CD19^+^ B cells, **B** CD69-expressing B cells, and **C** PD-L1-expressing B cells. **D** absolute numbers of CD19^+^ B, CD4^+^ and CD8^+^ T cells in total spleen cells. **E** Proliferation in CFSE-labeled CD4^+^ T cells in a Bdep: tBreg co-culture. **F** CD4^+^Foxp3^+^ Treg cells. Tumor burden and T cell responses in B-depleted TBM. B cells were depleted in mice three days before tumor challenge. B cells purified from NT or TBM were adoptively transferred to B-depleted TBM on days 3, 10, and 17 of tumor challenge. **G** tumor growth kinetics. **H** Bar diagram of T cell proliferation from different treatment groups (*n* = 6). **I** Representative flow dot plots of CD4^+^Foxp3^+^ Treg cells from different treatment groups. **J** Bar diagram of Treg cells (*n* = 6). Tumor generation experiments were repeated at least thrice (*n* = 4–6 at each repeat).

To better understand the mediators of tBreg production, we studied the changes in B and T cells over the time course of tumor progression in WEHI-164 tumor-bearing mice (TBM). The proportion of CD19^+^ B cells was consistently higher in TBM as compared to non-tumor controls (Fig. 2A). These B cells also expressed the activation marker CD69 in the late phases of tumor progression (Fig. 2B). Similarly, immunosuppressive PD-L1-expressing B cells increased as the tumor grew (Fig. 2C). The observed increase in CD19^+^CD69^+^ cells and CD19^+^PD-L1^+^ cells in TBM is consistent with our prior findings [19]. In contrast, the percentages of splenic CD4⁺ and CD8⁺ T cells significantly decreased at later time points (Fig. S2A, B, supporting information). To further confirm this decrease, we calculated the absolute number of CD19^+^ B and CD4⁺ and CD8⁺ T cells per spleen from NT and TBM. The absolute numbers of T cells from NT and TBM were unchanged, and the decrease in the T cells was due to the increased B cell proportion in TBM (Fig. 2D). To determine whether this increase in B cells is related to immune suppression, we performed a previously established T cell suppression assay [19]. As the tumor progressed, B cells progressively became immunosuppressive. By day 10, when tumors became palpable, TBM-B cells significantly suppressed T cell proliferation (Fig. 2E). In addition, as the tumor progressed, TBM displayed a marked increase in the frequency of CD4⁺Foxp3⁺ regulatory T cells (Tregs) (Fig. 2F).

To validate the role of tBregs in tumor immunosuppression, we depleted the B cells in mice with anti-CD19 antibody prior to WEHI-164 tumor cell inoculation. Effective B cell depletion was confirmed on the day of tumor challenge, without affecting the frequencies of T cells (Fig. S2C, supporting information). To further validate the role of B cells in immune suppression, purified B cells from either normal mice (B-NT) or WEHI-164 tumor-bearing mice (Breg) were adoptively transferred to B-depleted TBM on days 3, 10, and 17 following tumor cell inoculation. Tumor growth was significantly reduced in B-depleted mice, as compared to TBM (Fig. 2G). Adoptive transfer of both B-NT and Bregs resulted in a significant increase in tumor burden in B-depleted TBM (Fig. 2G). There was no significant change in tumor volume between TBM and B-NT or Breg-adoptively transferred Bdep TBM. On the other hand, the tumor burden of Breg-adoptively transferred Bdep TBM was significantly higher as compared to B-NT-adoptively transferred Bdep TBM (Fig. 2G). The increased tumor burden in B-NT-adoptively transferred Bdep TBM may be due to the conversion of B cells into Bregs in the presence of the tumor, which requires further validation to confirm. On day 20, the percentages of CD4^+^ T and CD8^+^ T cells were lower in TBM and improved in B-depleted TBM (Fig. S2D, supporting information). B cell frequency was increased in the TBM but remained low in B-depleted TBM even up to day 20 (Fig. S2E, supporting information). Re-expression of CD19 in the spleens of B-cell-adoptively transferred Bdep TBM confirms the presence of the transferred B cells even at the end of the experiment (Fig. S2E, supporting information). We next validated the effect of B depletion and adoptive transfer on T cell responses. Anti-CD3/CD28-induced proliferation was inhibited in CD4^+^ T cells from TBM but was significantly restored in B-depleted TBM, indicating a recovery of T cell function in the absence of B cells (Figs. 2H and S2F, supporting information). This increase in T cell proliferation was abrogated in B-NT and Breg-adoptively transferred Bdep TBM (Figs. 2H and S2F, supporting information**)**. The frequency of regulatory T cells was significantly higher in the TBM but not in the B-depleted TBM, and increased to levels similar to those in the TBM in B cell-adoptively transferred B-dep TBM. These data suggest a B-cell-dependent expansion of Tregs during tumor progression (Fig. 2I, J).

### PGE2-EP2/EP4 signaling induces tBreg-mediated suppression via STAT3 activation

**Fig. 3:**
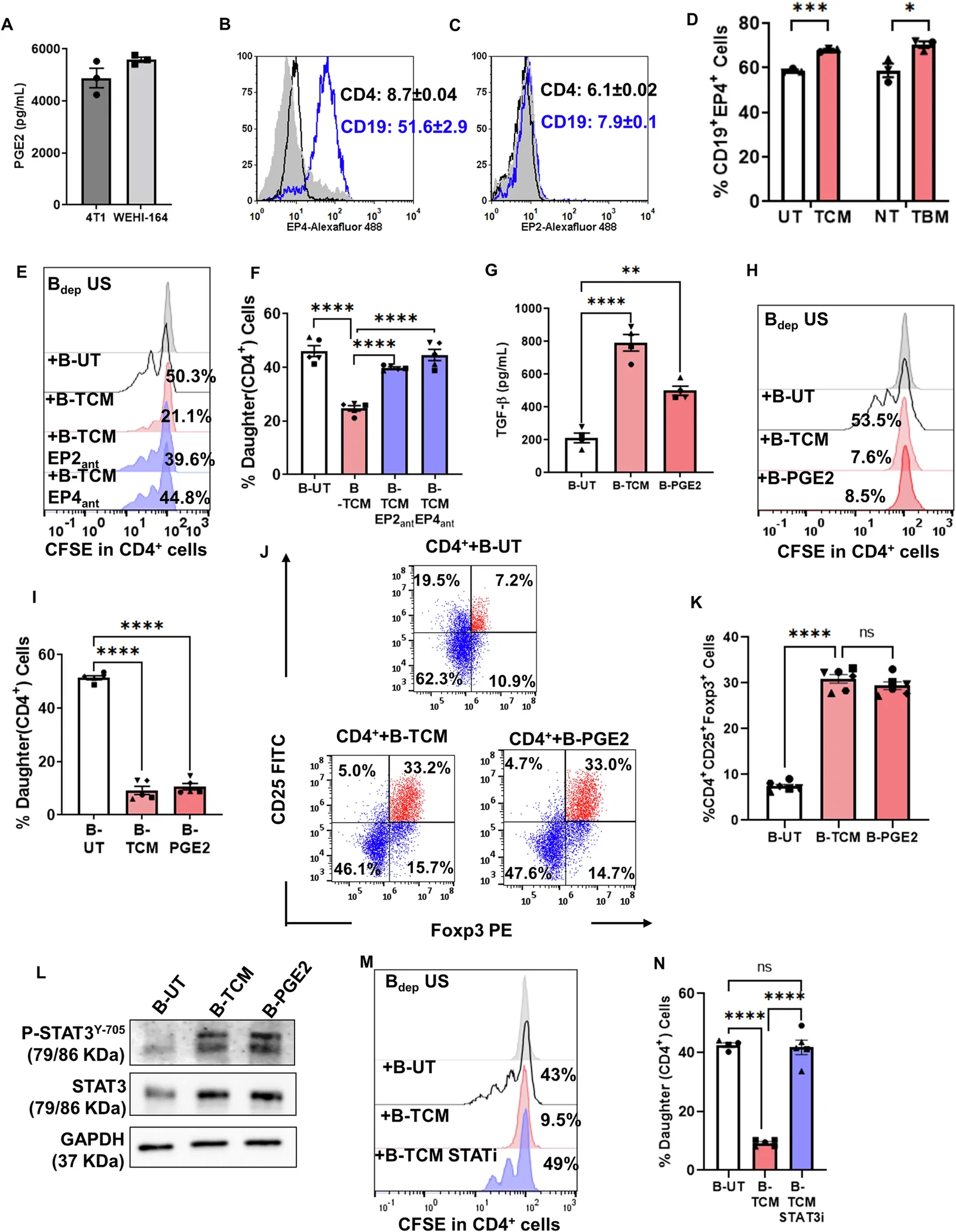
PGE2-EP2/EP4 signaling promotes tBreg-mediated suppression via STAT3 activation. **A** PGE2 in the supernatants of WEHI-164 and 4T1 cultures. PGE2 receptors **B** EP4 and **C** EP2 expression in CD4^+^ and CD19^+^ cells. **D** EP4 expression in CD19^+^ cells in the presence of tumor in vitro and in vivo. T cell responses with tBregs and EP antagonists. B cells were cultured with 20% TCM (B-TCM) along with EP2 and EP4 antagonists for 72 h and then co-cultured with CFSE-labeled, stimulated B_dep_ cells. **E** representative flow histograms of CFSE dye dilution in CD4^+^ gated T cells from different treatment groups. **F** Bar diagram of T cell proliferation (*n* = 5). Characterization of B-TCM and B-PGE2 tBregs. Purified B cells were cultured with 20% TCM or 1 μM PGE2 followed by a T cell suppression assay and Treg generation assay. **G** Secretion of TGFβ1 by B-TCM and B-PGE2 cells. **H** Representative flow histograms of CFSE dye dilution in CD4^+^ gated T cells from different treatment groups. **I** Bar diagram of T cell proliferation (*n* = 5). **J** Representative dot plots showing CD4^+^CD25^+^Foxp3^+^ Tregs from different treatment groups (**K**) Bar diagram of Treg cells (*n* = 7). Role of STAT3 signaling in tBregs: **L** P-STAT3 expression in B-TCM and B-PGE2 cells. B cells were pre-treated with the STAT3 inhibitor BP-1-102 and cultured with TCM, followed by a T cell suppression assay. **M** Representative CFSE histograms of CD4^+^ gated T cell proliferation during co-culture with tBregs and STAT inhibitor and **N** Bar diagram representation of T cell proliferation (*n* = 5).

We identified the tumor-derived soluble factor as a heat-stable, protease-resistant molecule with a molecular weight of less than 5 kDa (data not shown), and these characteristics are consistent with the lipid signaling mediator, prostaglandins (~350 Da). Prostaglandin E2 (PGE2), a prostanoid produced by the action of cyclooxygenase on arachidonic acid, has been shown to impair host immunity in several cancers [24–27]. Therefore, we measured PGE2 levels in WEHI-164 TCM, as well as 4T1, a previously known PGE2-secreting cell line [28]. WEHI-164 produced very high levels of PGE2 at ~4 ng/mL of TCM, comparable to those secreted by 4T1 (Fig. 3A).

PGE2 binds to membrane receptors EP1-4 to trigger signaling in target cells. Splenic CD19^+^ B cells had a significantly higher EP4 expression when compared to CD4^+^ T cells (Fig. 3B). While over 90% of CD19^+^ cells expressed the EP4 receptor, both T and B cells had low levels of EP2 expression, with B cells having slightly more EP2 than T cells (Fig. 3C). In addition, EP4 expression in CD19^+^ B cells increased with either in vitro TCM treatment or in the B cells from tumor-bearing mice (TBM) (Fig. 3D). Whereas EP2 expression in CD19^+^ B cells was either unchanged or decreased in the presence of the tumor (Fig. S3A, supporting information).

To further understand the role of EP receptors, tBregs were generated with TCM (B-TCM) in the presence of EP2 and EP4 neutralizing antibodies or potent EP2 (PF04418948) and EP4 (MK2894) antagonists. Neutralizing antibodies (B-TCMαEP2 and B-TCMαEP4) partially abrogated (Fig. S3B, C, supporting information), whereas both antagonists PF04418948 (B-TCMEP2_ant_) and MK2894 (B-TCMEP4_ant_) (Fig. 3E, F) completely reversed the B-TCM-induced suppression of CD4^+^ cell proliferation. Also, B cells treated with the agonists (PF-04217329, B-EP2_ag_, and CY10598, B-EP4_ag_) significantly suppressed T cell proliferation with an effect similar to B-TCM (Fig. S3D, E, supporting information). Collectively, our findings demonstrate that tumor-induced Breg generation is mediated by EP receptor signaling in B cells.

To confirm the effect of PGE2, purified B cells were treated with synthetic PGE2 (10 µM) (B-PGE2), and several WEHI-164-induced tBreg markers were examined [19]. In contrast to TCM, PGE2 did not increase the expression of tBreg-associated markers CD69 or PD-L1 (Fig. S3F, G, supporting information). B-PGE2 secreted significantly larger levels of TGF-β, albeit lower than B-TCM (Fig. 3G). B-PGE2 cells secreted IL-6 similar to B-TCM but did not secrete IL-10 in contrast to B-TCM (Fig S3H, supporting information). Similar to B-TCM, B-PGE2 effectively suppressed the proliferation of CD4^+^ cells, with comparable levels of suppression observed between the two groups (Fig. 3H, I). These cells were also able to generate Treg cells in a CD4^+^-B cell co-culture system (Fig. 3J, K).

Since we previously found increased STAT3 phosphorylation in B-TCM cells [19], we investigated if this was due to PGE2 signaling. Both B-TCM and B-PGE2 elevated STAT3 phosphorylation, as shown by Western blot and flow cytometry (Figs. 3L and S3I, J, supporting information). Decrease of STAT3 phosphorylation with α-EP4 neutralizing antibody in B-TCM confirmed that PGE2 signaling regulated STAT3 phosphorylation (Fig. S3K, supporting information).

To validate PGE2-mediated STAT3 phosphorylation in tBregs, we inhibited STAT3 phosphorylation in B-TCM cells using the STAT3 inhibitor BP-1-102 (Fig. S3L, supporting information). BP-1-102 treatment resulted in a complete restoration of B-TCM-induced suppression of responder T cell proliferation (Fig. 3M, N). Collectively, these results indicate that PGE2-induced tBreg generation was facilitated by STAT3 activation.

### PGE2-induced STAT3 phosphorylation in Bregs is mediated through non-canonical MAPK signaling

**Fig. 4:**
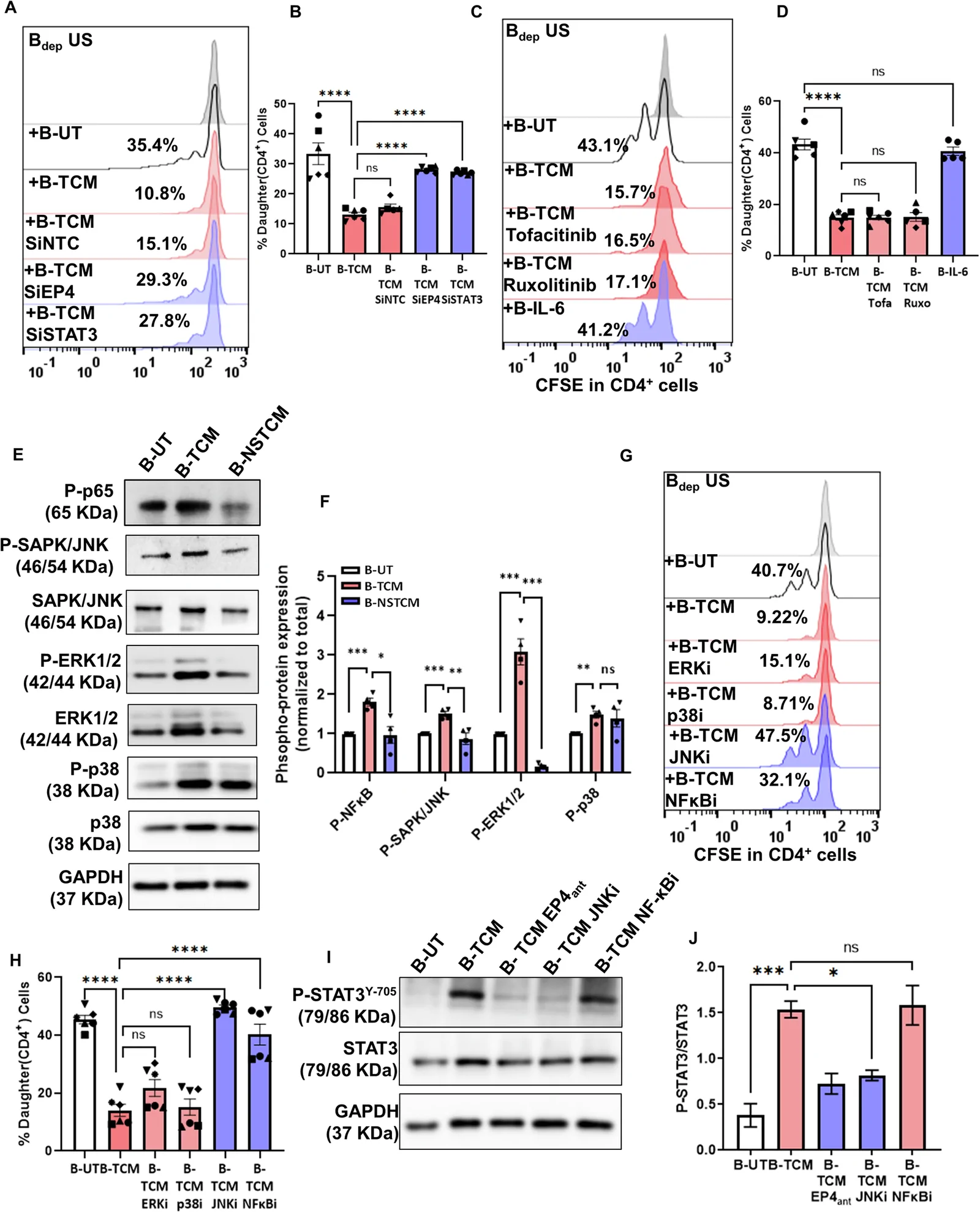
PGE2 induces STAT-3 phosphorylation in Breg via non-canonical MAPK signaling. Effect of EP4 and STAT3 siRNAs on Bregs. Purified B cells were transfected with si RNAs against EP4, STAT3 and non targeting control (siEP4, siSTAT3 and siNTC) for 24 h. The transfected cells were treated with 20% TCM to generate Breg cells, followed by a T cell suppression assay. **A** Representative histograms of CD4^+^ gated T cell proliferation in different treatment groups. **B** Bar diagram of T cell proliferation (*n* = 6). Effect of IL-6 signaling on Bregs. B cells were either pre-treated with IL-6 signaling inhibitors Ruxolitinib and Tofacitinib and cultured with 20% TCM or 20 ng/mL recombinant mouse IL-6, followed by a T cell suppression assay. **C** Representative histograms of CD4^+^ gated T cell proliferation in different treatment groups. **D** Bar diagram of T cell proliferation (*n* = 6). **E** Western blot images and **F** Quantification of NF-kB and MAPK protein expression in tBregs treated with TCM and NS-TCM (*n* = 3). Effect of non-canonical protein inhibitors of Bregs. B-TCM were generated in the presence of inhibitors against ERK, p38, SAPK/JNK, and NFκB (10 μM), followed by a T cell suppression assay. **G** Representative histogram of CD4^+^ gated T cell proliferation in different treatment groups. **H** Bar diagram of T cell proliferation (*n* = 6). **I** Western blot of P-STAT3 in tBregs treated with inhibitors and **J** quantitation of P-STAT3/STAT3 (*n* = 3).

To further confirm the involvement of PGE2/STAT3 signaling in Breg cell induction, we knocked down EP4 and STAT3 expression in B cells using RNA interference prior to Breg induction (Fig. S4A, B, supporting information). We selected EP4 since it is more abundant in B cells and increases with tumor conditions rather than EP2. Both SiEP4 and SiSTAT3 abrogated tBreg generation and its T cell suppressive function (Fig. 4A, B). Silencing of EP4 also decreased STAT3 phosphorylation, while STAT3 silencing did not affect EP4 expression (Fig. S4A, B, supporting information). These data confirm that STAT3 activation is indeed downstream of PGE2/EP4 signaling. We next sought to identify the downstream pathway leading to STAT3 activation in Breg cells.

IL-6, a classical upstream activator of STAT3, was elevated in both B-TCM and B-PGE2 cells. To mechanistically delineate the role of IL-6 in STAT3 activation, we either blocked IL-6 signaling in Bregs with a JAK1/2 inhibitor, Ruxolitinib, and a JAK1/3 inhibitor, Tofacitinib, or treated B cells with recombinant mouse IL-6. Inhibition of IL-6 signaling did not reverse Breg immunosuppressive function (Fig. 4C, D). Similarly, purified B cells treated with recombinant mouse IL-6 (B-IL-6) did not acquire immunosuppressive properties and failed to suppress T cell proliferation (Fig. 4C, D). These results showed that the tBreg phenotype was independent of IL-6 and may be mediated by non-canonical STAT3 activation.

NS-398, a selective cyclooxygenase 2 (COX2) inhibitor, was used to block PGE2 secretion by WEHI-164 cells [29]. This NS-TCM was used to identify the non-canonical MAPK signaling events associated with tBreg generation. NF-kB and MAPK proteins JNK and ERK were hyperphosphorylated in B-TCM and returned to basal levels in B-NSTCM cells (Fig. 4E, F). To identify the individual role of these pathways, B cells were pre-treated with selective pharmacological inhibitors—NF-κB activation inhibitor II (B-NF-κBi), SP600125 (B-JNKi), UO126 (B-ERKi), and SB203580 (B-p38i)—prior to TCM treatment (Fig. S4C, supporting information). The ERK1/2 inhibitor or p-38 inhibitor did not have any effect on the tBreg-induced inhibition of T cell proliferation (Fig. 4G, H). Both JNK and NF-κB inhibitors completely abrogated tBreg-mediated T cell suppression (Fig. 4G, H). Notably, only JNK inhibition, but not NF-κB inhibition, decreased STAT3 activation in B cells (Fig. 4I, J). These data suggest that PGE2/EP4-induced JNK activation mediates STAT3 phosphorylation in Bregs and regulates Breg immunosuppressive function. PGE2/EP4-induced NF-κB activation did not have a direct role in STAT3 activation but regulated Breg immunosuppressive function. Both the transcription factors STAT3 and NF-κB could be independently activated downstream of PGE2/EP4 signaling, and many forms of crosstalk between these two proteins exist, including physical interaction and cooperation at promoters/enhancers [30]. Further studies are therefore required to identify the interplay between JNK, STAT3, and NF-κB in tBreg induction and function in fibrosarcoma.

### The COX-2 inhibitor NS-398 reduced tumor burden by reversing Breg-mediated T cell suppression

**Fig. 5:**
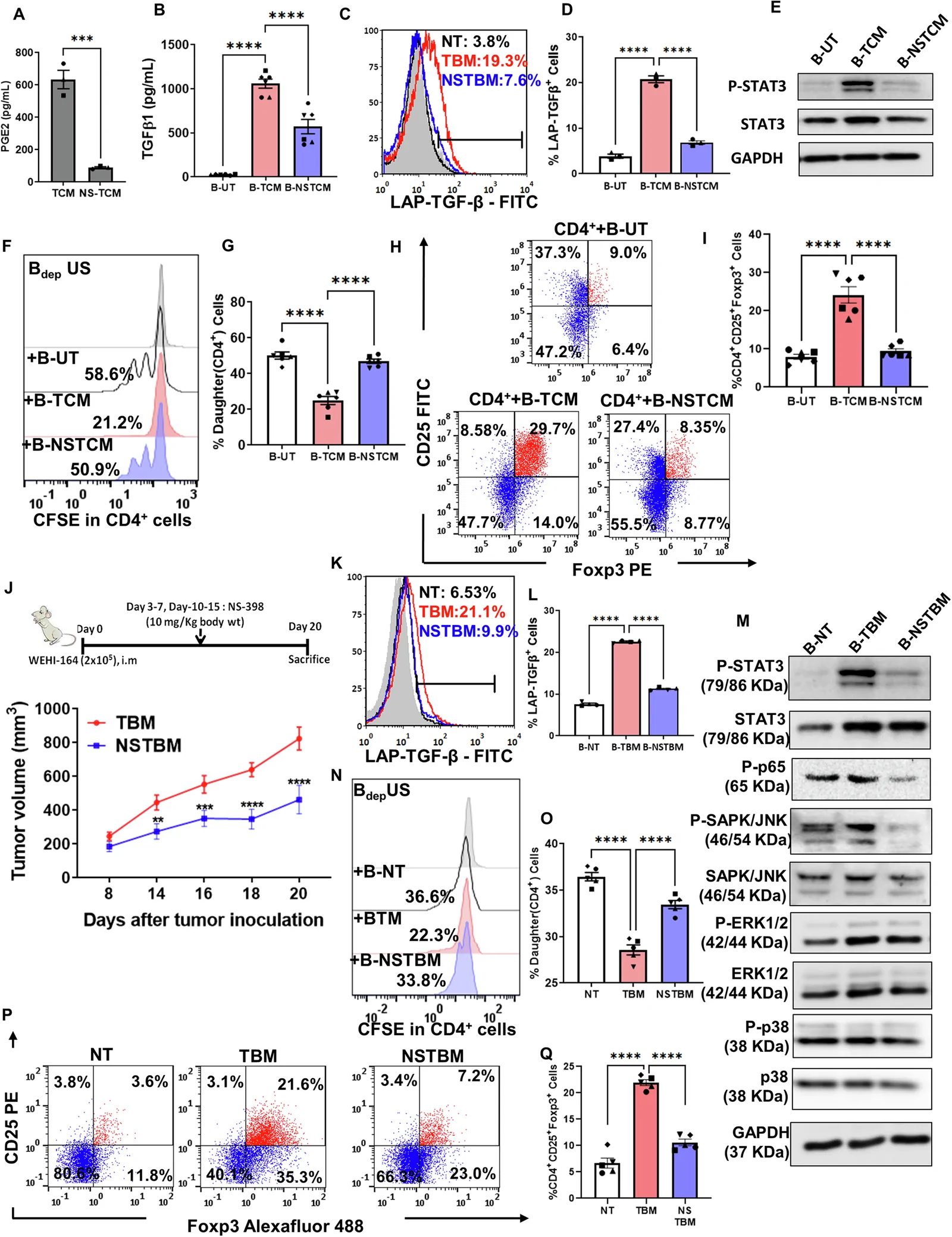
NS-398 reduced tumor burden by reversing Breg-mediated T cell suppression. **A** PGE2 levels in TCM and NS-TCM. Effect of NS-TCM on tBreg generation and function. B cells were cultured with 20% TCM or NS-TCM. **B** TGF-β1 production by B-TCM and B-NSTCM cells. **C** Representative flow histogram and **D** Bar diagram of membrane-bound TGF-β expression (*n* = 3). **E** P-STAT3 Western blot. **F** Representative histogram of CD4^+^ gated T cell proliferation cultured with B-TCM and B-NSTCM. **G** Bar diagram of T cell proliferation (*n* = 6). **H** Treg generation upon co-culture with B-TCM and B-NSTCM and **I** bar diagram of Treg cells (*n* = 7). NS398 lowers tumor burden through reduced Breg and Treg. NS-398 (10 mg/kg body weight) was administered in WEHI-164 TBM for 10 days from day 3 of tumor inoculation. On day 20, the mice were sacrificed, and spleens were isolated to assess T and B cells. **J** tumor volume. **K** Representative flow histograms and **L** Bar diagram of TGF-β expressing B cells from TBM and NSTBM (*n* = 4). **M** Expression of signaling proteins in B cells purified from TBM and NS-TBM. **N** Representative histograms and **O** Bar diagram of CD4^+^ gated T cell proliferation during co-culture with B cells from TBM and NS-TBM (*n* = 5). **P** Dot plots and **Q** Bar diagram of Treg generation in spleen cells from TBM and NS-TBM (*n* = 5). Tumor generation experiments were repeated at least thrice (*n* = 4–6 at each repeat).

Since there was abrogation of B-TCM-induced phosphorylation of the non-canonical STAT3 activators in B-NSTCM, we further evaluated whether NS-398 administration in tumor-bearing mice could lead to functional restoration of B cells. NS-398 was non-cytotoxic to WEHI-164 cells at concentrations up to 100 µM (Fig. S5A, supporting information), and NS-398 induced a substantial reduction in PGE2 release even at 10 μM (Fig. 5A).

B-NSTCM produced significantly lower levels of TGF-β (Fig. 5B), IL-6, and IL-10 (Fig. S5B, supporting information). B-NSTCM showed a significant reduction in the percentage of membrane-bound TGF-β (LAP-TGFβ) (Fig. 5C, D). In addition, the CD19^+^CD81^+^CD27^+^ population of B-NSTCM cells decreased significantly when compared to B-TCM cells (Fig. S5C, supporting information). CD86 expression was marginally lower in B-NSTCM cells (Fig. S5C, supporting information). However, other WEHI-164 tBreg markers such as CD40, CD80, IA/IE^hi^, and PD-L1 were equivalent in B-NSTCM cells compared to B-TCM tBregs (Fig. S5C, supporting information). P-STAT3 levels were significantly reduced in B-NSTCM cells compared to B-TCM tBregs (Figs. 5E and S5D, supporting information). B-NSTCM significantly improved tBreg-mediated suppression of T cell proliferation (Fig. 5F, G). The B-NSTCM cells also failed to generate Treg cells, unlike B-TCM cells (Fig. 5H, I), confirming that tBregs were generated by PGE2 signaling.

To confirm that tumor-secreted PGE2 is solely responsible for tBreg generation, we inhibited the other arachidonic acid metabolic intermediates in WEHI-164 cells, resulting in the generation of corresponding TCMs. We suppressed leukotriene generation by inhibiting 5-lipoxygenase with zileuton and thromboxane generation with the thromboxane-A_2_ inhibitor dipyridamole [31, 32]. Unlike NS-TCM, B cells generated with zileuton- and dipyridamole-treated TCM did not rescue T cell proliferation in a co-culture assay, confirming that PGE2 is the key mediator of immunosuppressive tBreg induction by the WEHI-164 tumor (Fig. S5E, supporting information).

Administration of NS-398 significantly decreased tumor growth when compared to TBM (Fig. 5J), along with an increase in CD4^+^ cells and CD8^+^ cells (Fig. S5F, supporting information) and no change in CD19^+^ B cells (Fig. S5F, supporting information). However, NS-398 treatment significantly reduced TGF-β-expressing B cells in TBM (Fig. 5K, L). TBM tBregs exhibited increased phosphorylation of STAT3, NFκB, SAPK/JNK, and ERK1/2 compared to B cells from NS-398-treated TBM (Figs. 5M and S5G, supporting information), supporting our in vitro findings that NS-398 treatment prevents the development of the tBreg phenotype. B cells from vehicle-treated TBM inhibited T cell proliferation, but B cells from NS-398-TBM significantly improved T cell responses ex vivo (Fig. 5N, O). The percentage of Treg cells that increased in vehicle-TBM decreased significantly after NS-398 treatment, showing that the Breg-Treg axis was disrupted (Fig. 5P, Q).

To confirm that tumor-derived PGE2 and tBregs do actually cause immunosuppression, we administered NS-398 to B-depleted TBM. NS-398 treatment and B-depletion reduced tumor development individually but not in combination (Fig. S6A, supporting information). The percentages of Treg cells in the NS-Bdep TBM were comparable to those of the individual treatments (Fig. S6B, C, supporting information). The proliferation efficiency of splenic T cells was also evaluated. While NS-398 and B-depletion greatly improved T cell proliferation, combining the two did not further improve T cell responses (Fig. S6D, E, supporting information). These findings show that COX-2 inhibition with NS-398 reduced tumor-derived PGE2. This in turn blocked tBreg generation, disrupted the regulatory B-T axis, relieved tumor-induced immunosuppression, and restored T cell responses, lowering the tumor burden in preclinical models. Since there were no B cells in B-depleted mice, there was no further improvement because of NS-398 treatment.

### TGFB1-producing Bregs are present in human sarcomas and other cancers with high PGE2

**Fig. 6:**
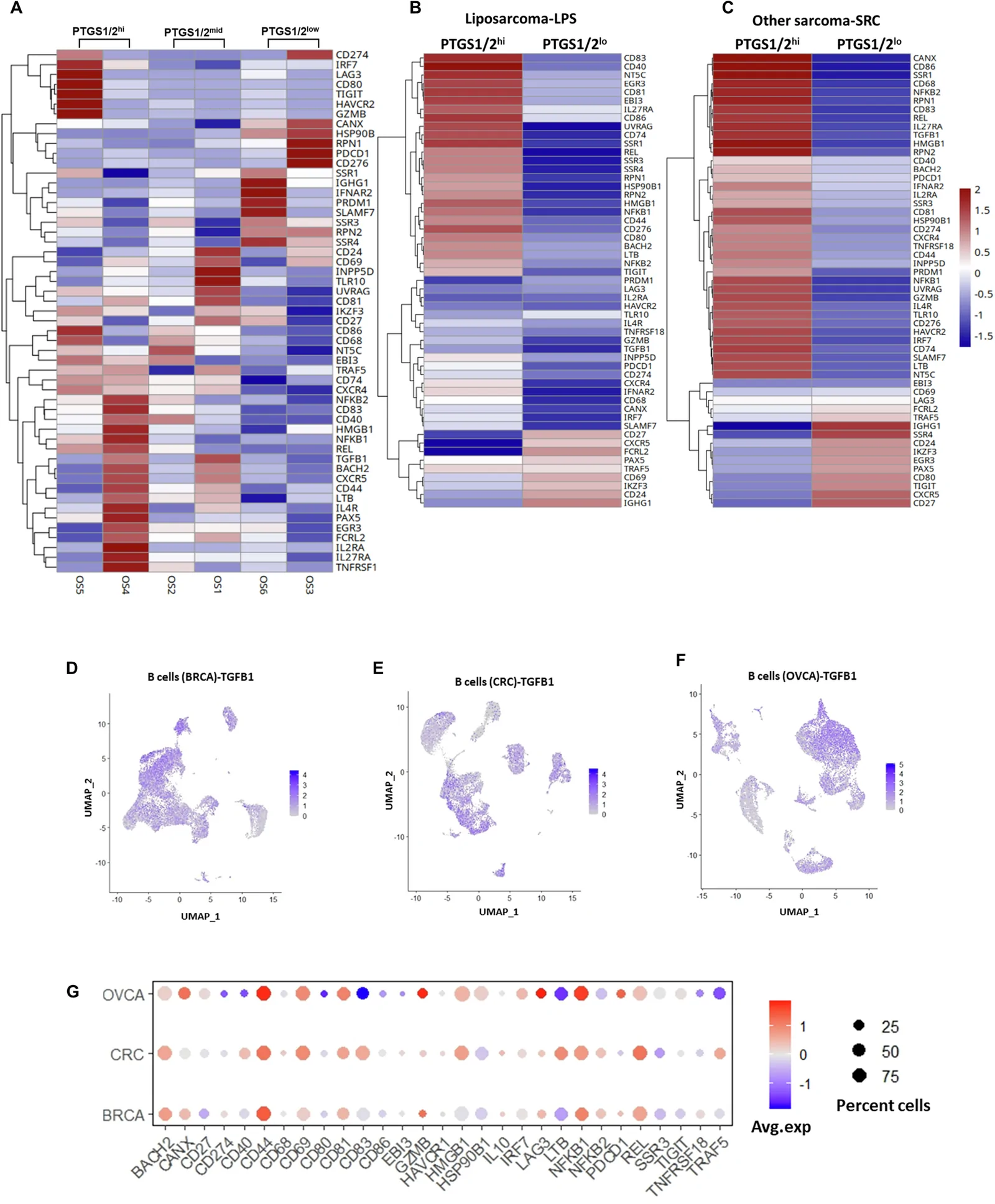
TGF-β-producing Bregs are present in human cancers with high PGE2 levels. Publicly available single-cell transcriptomics data from human cancers (osteosarcoma, LPS, SRC, BRCA, CRC, and OVCA) were analyzed for Breg cells. ScRNAseq samples from OS, LPS, and SRC were categorized into PTGS1/2^hi^ and PTGS1/2^lo^ based on the expression of *PTGS1* and *PTGS2*. B cell subset and expression of common Breg markers were assessed. **A** A heatmap showing relative (z-scored per row) gene expression levels of Breg genes among individual OS samples arranged in order of PTGS1/2 hi to low. **B** Breg genes in B cells from PTGS1/2^hi^ and PTGS1/2^lo^ LPS. **C** Breg genes in B cells from PTGS1/2^hi^ and PTGS1/2^lo^ SRC. B cells were filtered from ScRNA-seq samples of CD45^+^ TILs from high PTGS2-expressing human BRCA, CRC, and OVCA samples. *TGFB1* expression in B cell subclusters of **D** BRCA, **E** CRC, and **F** OVCA. Filtered B cells were grouped into TGF-β^lo^ and TGF-β^hi^ B cells. **G** bubble plot showing Breg genes in TGF-β^hi^ B cells from BRCA, CRC, and OVCA.

To obtain an insight into PGE2/EP4 signaling-mediated regulatory B cell induction in human cancers, we first evaluated whether such a condition is prevalent in the osteosarcoma (OS) samples using the single-cell transcriptomic data. We first mapped the expression of the *PTGS1* and *PTGS2* genes that encode for COX1 and COX2 enzymes in individual OS samples (Fig. S7A, supporting information) and grouped OS3 and OS6 as PTGS1/2^lo^, OS1 and OS2 as PTGS1/2^mid^, and OS4 and OS5 as PTGS1/2^hi^ samples. EP4 receptor (*PTGER4*) in B cells from these samples showed increased expression in PTGS1/2^hi^ samples (Fig. S7B, supporting information). Breg-associated gene expression analysis in B cells from these groups showed increased *TGFB1* and Breg signature in PTGS1/2^hi^ samples and PTGS1/2^mid^ samples as compared to PTGS1/2^lo^ samples. Most of the Breg genes were highly upregulated in PTGS1/2^hi^ samples. Interestingly, NF-κB pathway genes (*NFKB1*, *NFKB2*, *REL*, and *TRAF5*) were also upregulated in PTGS1/2^hi^ and PTGS1/2^mid^ B cells, similar to that of *TGFB*^hi^ Breg cells, indicating that B cells from PTGS1/2-expressing cancers indeed show the Breg phenotype (Fig. 6A).

We then asked whether B cells exhibit a similar Breg phenotype in other human sarcomas with high prostaglandin levels. We utilized publicly available single-cell transcriptomic data of soft tissue sarcoma [33]. We first calculated B cell frequencies in human liposarcoma (LPS, *n* = 10), undifferentiated pleomorphic sarcoma, and mixofibrosarcoma (SRC, *n* = 7). Overall, B cell infiltrations were lower in LPS and SRC (Fig. S7C, D, supporting information). We then evaluated the expression of *PTGS1/2* in individual samples and grouped them as PTGS1/2^hi^ and PTGS1/2^lo^ based on expression (Fig. S7E, F, supporting information). B cells from each group were then filtered, and Breg markers were assessed. Similar to OS, common Breg-associated markers showed increased expression in PTGS1/2^hi^ LPS and SRC (Fig. 6B, C). Genes like NF-κB pathway genes (*NFKB1*, *NFKB2*, and *REL*), *HSP90B1*, *HMGB1*, *LTB*, *CD81*, and *CD83* were among the commonly upregulated genes in LPS and SRC. TGFB1 was also higher in PTGS1/2^hi^ B cells in LPS and SRC, but the extent of increase was more evident in SRC as compared to LPS (Fig. 6B, C).

We next investigated whether this is a sarcoma-specific phenomenon or whether such Bregs can be induced in other human cancer types expressing high levels of PGE2. For this, we utilized single-cell transcriptomic data of tumor-infiltrating CD45^+^ immune cells from breast cancer (BRCA; *n* = 5), ovarian cancer (OVCA; *n* = 5), and colorectal cancer (CRC; *n* = 5) previously published by Punyawatthananukool et al. [34]. They have identified that these cancer tissues expressed high levels of PGE2 through both COX1 and COX2 via immunohistochemistry [34]. We first enumerated the percentages of immune cells in individual samples (Fig. S7G–I, supporting information). Most of the samples showed B-cell infiltration, and we selected samples with higher B-cell infiltration within each cancer type (BRCA_2, BRCA_4, CRC_1, CRC_3, CRC_4, OVCA_1, OVCA_2, and OVCA_3) for further analysis. B cells from each type were pooled within, subclustered, and checked for the expression of *TGFB1*. Most of the B cell clusters showed increased *TGFB1* in all three cancer types, indicating the presence of *TGFB1-*producing Breg cells (Fig. 6D–F). We then grouped the B cells as TGF-β^lo^ and TGF-β^hi^ B cells based on the median expression of *TGFB1* in each cancer type. The TGF-β^hi^ B cells also showed increased expression of common Breg markers (Fig. 6G). Similar to OS, LPS, and SRC B cells, TGF-β^hi^ B cells from BRCA, CRC, and OVCA showed increased expression of NFκB pathway genes (*NFKB1*, *NFKB2*, and *REL*). These data, along with our experimental results, suggest that tumor-derived PGE2 induces TGF-β-secreting Breg cells, which are regulated at least partially by NF-κB signaling.

### A high Breg profile is associated with increased immunosuppressive markers and poor survival in PTGS2-high cancer patients

**Fig. 7:**
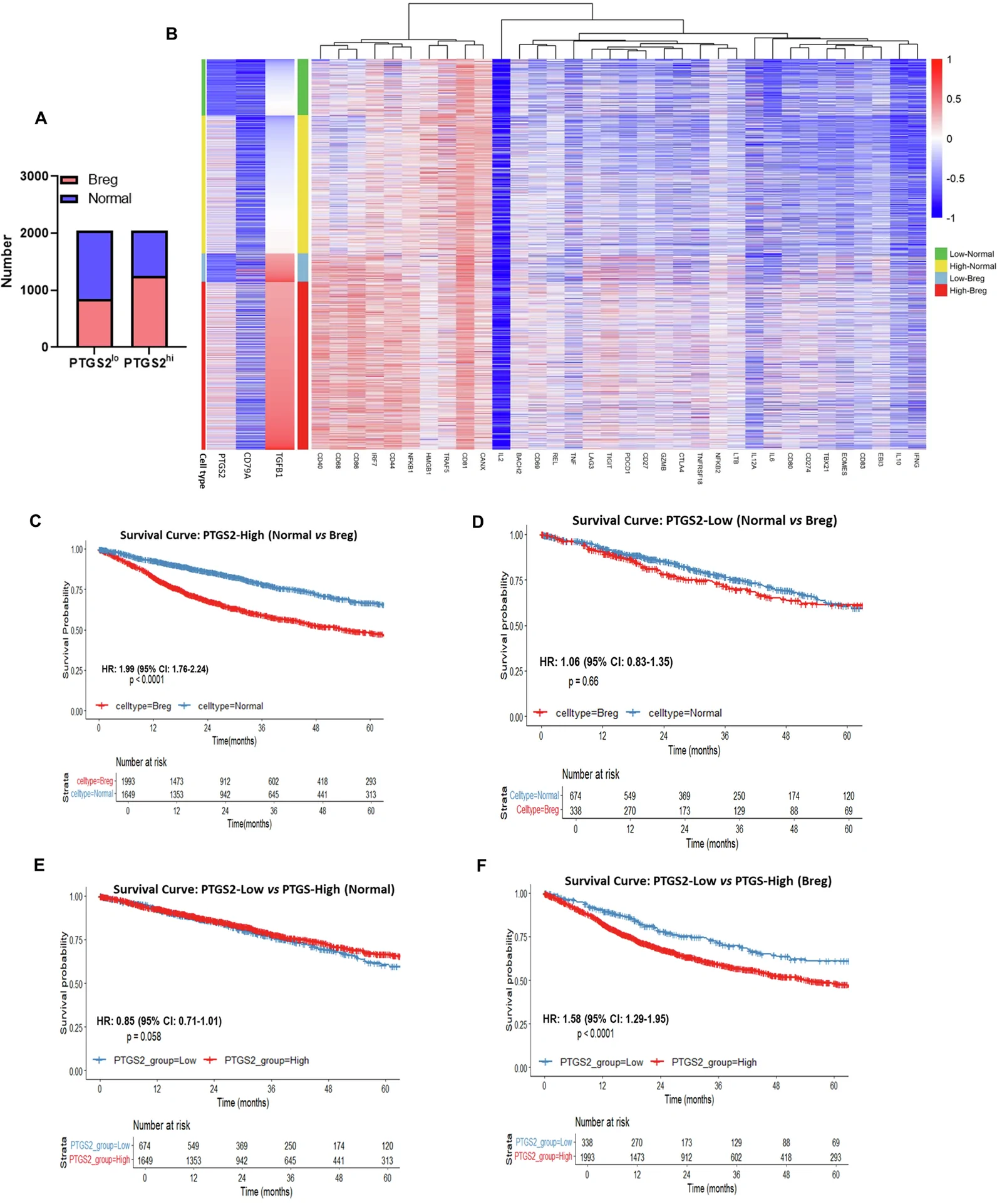
Breg Signature is associated with poor survival in PTGS2-high cancer patients. TCGA-Pancancer samples were classified into PTGS-high and PTGS2-low based on the survival cutoff of *PTGS2* expression. The “Breg” signature was defined as strong B cell marker (*CD79A*) expression along with high *TGFB1* expression (>Q3), whereas the “Normal” signature was defined as strong B cell marker (*CD79A*) expression along with low *TGFB1* expression (<Q1). **A** Number of samples showing “Normal” and “Breg” frequency in PTGS-2^lo^ and PTGS-2^hi^ patients. The patient samples were classified into “Low-Normal,” “High-Normal,” “Low-Breg,” and “High-Breg.” **B** Co-expression of immunosuppressive genes in “PTGS2 Low-Normal,” “PTGS2 High-Normal,” “PTGS2 Low-Breg,” and “PTGS2 High-Breg” samples. **C** Survival curve comparing patients with PTGS2 High-Normal (blue) and PTGS2 High-Breg (red). **D** Survival curve comparing patients with PTGS2 Low-Normal (blue) and PTGS2 Low-Breg (red). **E** Survival curve comparing patients with PTGS2 Low-Normal (blue) and PTGS2 High-Normal (red). **F** Survival curve comparing patients with PTGS2 Low-Breg (blue) and PTGS2 High-Breg (red). The threshold of the COX *P* value is < 0.05.

A wide range of human cancers have high COX2 levels, which result in high PGE2 levels. Comparison of *PTGS2* expression in different cancer types from the TCGA pan-cancer bulk RNA-seq data is given in Fig. S8A, supporting information. To further explore the relevance of the TGFβ-Breg cell subpopulation in COX2-expressing cancers, we deconvoluted the TCGA pan-cancer bulk RNA-seq data using canonical B cell markers *MS4A1* and *CD79A* (log2TPM ≥ 2.58), representing samples with strong B cell infiltration. We then further stratified this B-cell-enriched cohort into PTGS2^hi^ and PTGS2^lo^ according to the optimal survival-based cutoff of *PTGS2* expression. Based on the quartile expression of *TGFB*, we then classified the B cell cohort as “Normal” (TGFB < Q1) and “Breg” (TGFB > Q3). The frequency of the Breg profile was higher in patients with high PTGS2 expression (Fig. 7A). The frequency of patients with “Normal” and “Breg” profiles in different cancer types is shown in Fig. S8B, supporting information.

We then asked whether this Breg signature is also associated with immunosuppression. We checked the expression of common checkpoint markers and T cell-associated cytokines and checkpoint markers in the four classes: PTGS2^lo^: Normal (Low-Normal), PTGS2^hi^: Normal (High-Normal), PTGS2^lo^: Breg (Low-Breg), and PTGS2^hi^: Breg (High-Breg). Expression of immunosuppressive markers was compared amongst the PTGS2-^hi^ but normal to Breg populations (Fig. 7F). There was a clear upregulation of *TGFB1*, *PDCD1*, *LAG3*, *CTLA4*, *TIM3*, and *TIGIT*. Interestingly, an increase in Granzyme B was also observed. Since granzyme B was upregulated in B cells from PTGS2^hi^ cancers from our single-cell RNA sequence analyses, we assessed granzyme levels in fibrosarcoma Bregs. Granzyme B was increased in B cells treated with TCM and B cells from TBM (Fig. S9A, supporting information). However, this increase in granzyme B in tBreg cells did not induce any cytotoxicity in T cells in a B: T co-culture setup (Fig. S9B, C, supporting information). When these T cells from the co-culture were isolated and tested for cytotoxicity in target tumor cells, we observed a decrease in granzyme B secretion and cytotoxicity (Fig. S9D, E, supporting information), suggesting a possible exhaustion of T cells in the presence of tBregs.

Breg populations also clustered into PTGS2^lo^ and PTGS2^hi^, indicating the development of Bregs by a PGE2/EP4-independent pathway. Both these PTGS2^lo^ Breg and PTGS2^hi^ Breg cancers had increased expression of immunosuppressive markers like *TGFB1*, *PDCD1*, *LAG3*, *CTLA4*, *TIM3*, and *TIGIT*, indicating immune exhaustion. To confirm this, we assessed the expression of exhaustion markers like CTLA4, LAG3, PD1, and TIM3 in CD4^+^ and CD8^+^ T cells from WEHI-TBM. There was indeed an increased expression of these immunosuppressive markers in T cells from TBM (Fig. S9F, G, supporting information). Most of these markers were decreased in T cells from Bdep TBM, suggesting the role of Breg cells in inducing exhaustion in T cells (Fig. S9F, G, supporting information). Dependency of COX2/PGE2 signaling in Breg-induced T cell exhaustion was further confirmed by the decrease of these markers in T cells co-cultured with Breg cells generated with NS-TCM (Fig. S9H, supporting information). These findings also suggest that elevated GzmB expression in PTGS2^hi^ tumor conditions may not reflect enhanced cytotoxic T cell activity but may instead be associated with exhaustive or immunosuppressive states.

We next assessed whether the Breg signature and associated increase in immunosuppression markers have any implications for the overall survival of these patients. Survival analysis showed that patients with a “Breg” profile had significantly poorer overall survival than the patients with a “Normal” B cell profile in the PTGS2^hi^ group, whereas in the PTGS2^lo^ group, no difference was observed between “Normal” and “Breg” profiles (Fig. 7C, D). We also compared the survival status of PTGS2^hi^ versus PTGS2^lo^ in patients with “Normal” and “Breg” profiles. In patients with a “Normal” profile, the survival status was unaffected by the expression of *PTGS2* (Fig. 7E). In the case of patients with a “Breg” profile, the PTGS2^hi^ expression condition significantly decreased the survival probability (Fig. 7F). These data suggest that a combination of Breg signature and high *PTGS2* expression is related to poor prognosis.

## Discussion

The regulatory effect of B cells, first described in delayed-type hypersensitivity responses, has been characterized in autoimmunity but not well characterized in cancer. In this study, we demonstrate that TGF-β-producing Bregs are present in sarcomas and are associated with immunosuppression. Depletion of B cells in mice decreased fibrosarcoma tumor burden and improved T cell responses. These effects were reversed with the adoptive transfer of B cells in B-depleted TBM. These tumor-evoked Bregs were induced by a PGE2/EP4-mediated non-canonical STAT3 signaling mechanism. Inhibition of COX-2 in vitro or in vivo abrogated tBreg generation and reversed the immunosuppression. PGE2^hi^ TGF-β^hi^ Bregs were found in multiple sarcomas and other cancers and were associated with poor overall survival. These findings indicate that PGE2^hi^ TGF-β^hi^ Bregs play a crucial role in cancer progression and are potential therapeutic targets.

In osteosarcoma, there is a heterogeneity of B cell populations, and those with high TGF-β levels were associated with poor prognosis [35]. It is unclear whether this heterogeneity was due to Bregs, and no single surface or secreted marker defines Bregs. They can be induced by several signals, such as apoptotic cells, gut microbiota, BCR, TLR, CD40, and CD80/86 signaling. Equally complex are the mechanisms of immunosuppression employed by the Bregs that range from cytokines such as IL-10, IL-35, TGF-β, and Granzyme B to the checkpoint proteins like PD-L1, TIM3, LAG-3, TIGIT, and CD1d. Apart from directly suppressing the DCs, macrophages, or T cells, they can also induce Tregs that can, in turn, cause immunosuppression [36]. In our study, the immunosuppressive functions of WEHI-164-induced tBregs are mediated by the cytokine TGF-β1, primarily by suppressing T cell proliferation and converting naïve T cells to Treg cells. This finding is consistent with the established role of TGF-β1 in promoting regulatory T cell generation. Several researchers, including the seminal work by Zheng et al., demonstrated that TGF-β can drive the conversion of naïve CD4⁺CD25⁻ T cells into functional Foxp3⁺ regulatory T cells in both murine and human setups [37–40]. TGF-β1 is produced as an inactive precursor and requires activation. Activated TGF-β1 exerts its biological effects only after the cleavage of the latency-associated protein (LAP) and latent TGF-β binding protein (LTBP) from the large latent complex [41]. Measuring active TGF-β1 levels and localization is also important for understanding its secretion by tBregs. Furthermore, TGF-β receptor levels also influence the activation of signaling pathways and their biological effects [42].

In this study, we report for the first time that PGE2-EP2/EP4 signaling can lead to TGF-β-producing Bregs in mouse fibrosarcoma. We also show that TGF-β-producing Bregs are present in human sarcomas and other cancers with high PGE2. Prostaglandins are arachidonic acid metabolism products produced by the enzyme cyclooxygenase-2 (COX2), which plays a crucial role in inflammation and cancer [43]. COX2 is often overexpressed in a variety of human and murine cancers [44]. PGE2, the most abundant metabolite of the COX2 pathway, is responsible for the majority of COX-2-mediated immune suppression in the TME [43, 44]. PGE2 promotes tumor growth, angiogenesis, and metastasis, but it also reduces antitumor immunity by increasing the proportion of immunosuppressive immune cell types in the TME and decreasing T_H1_ and CD8^+^ responses [43]. tBregs have been shown to be induced by leukotriene B4 (LTB4), a lipid metabolite [20]; tumor-derived cytokines such as IL-18 and IL-21 [17, 45] or tumor-derived exosomes containing PD-L1 [46] or HMGB1 [47] payloads. In another study, MAP3K2 and RAP1B were identified as the hub genes involved in the production of IL-10^+^PD1^hi^ B10 Bregs from healthy peripheral blood CD19^+^ B cells [48].

The B lineage signature was found to be associated with better overall survival in sarcomas [5, 49]. However, these B cells were associated with tertiary lymphoid structures (TLS), and their TGF-β or PGE2 status is not clear. TLS are organized immune cell clusters formed in non-lymphoid structures, particularly in the tumor microenvironment, and have been associated with improved survival and immunotherapy response [50]. The role of PGE2 in TLS is not clear, but since PGE2 mediates a tolerogenic environment with DC and T cell dysfunction, it may not support the formation of TLS [28, 51]. Our results show that PGE2^hi^, TGF-β^hi^ cancers with Breg signatures have poor survival. They have very high expression of all the immune checkpoint markers and granzyme B.

In murine fibrosarcoma, we established the significance of PGE2-EP2/EP4 signaling in tBreg generation using EP2/EP4 agonists. In addition, neutralizing antibodies or EP2/EP4 antagonists inhibited PGE2-induced tBreg generation. These PGE2-induced tBreg cells produced Tregs and inhibited T cell proliferation in a B-T cell co-culture, validating our findings. TCM treatment increased the expression of B cell EP receptors in vitro, and a similar effect was observed in TBM tBregs. EP4 receptors play a key role in modulating inflammatory responses, and they have been shown to regulate their own expression in macrophages [52]. EP4 is a delayed early gene that is activated and works as a negative feedback regulator of B cell proliferation. PGE2 as low as 1 nM could inhibit B cell proliferation in activated cells [22].

We have previously shown that PGE2/EP4 signaling promotes the miR-365/IL-6/STAT3 pathway, which affects dendritic cells. However, in B cells, PGE2-EP2/EP4-induced STAT3 may not be through IL-6 and is mediated by a non-canonical signaling pathway. STAT3 regulates bioenergetics and metabolic pathways in B cells, and mice lacking STAT3 in CD19^+^ B cells had fewer Bregs and Tregs, leading to worsening autoimmune conditions [53, 54]. PGE2-induced STAT3 seems to be mediated through a non-canonical pathway, and the exact signaling mechanisms are not clearly understood [54–56]. Our studies demonstrate that this is through MAPK, particularly JNK signaling. PGE2/EP4/ERK-mediated STAT3 activation has been reported in cardiomyocytes [57].

The COX2 inhibitor NS-398 reduced PGE2 levels and also completely inhibited tBreg generation, as shown by the absence of T cell suppression. NS-398 inhibited the PGE2-induced IL-6, IL-10, and TGF-β secretion and STAT3 phosphorylation but only marginally reduced other Breg markers. Tumor-bearing mice treated with NS-398 showed similar effects of a decrease in Treg, tumor burden, and improved T cell response. In 4T1 tumor-bearing mice, this effect of NS-398 was mostly because of effective dendritic cells [28]. PGE2 also has direct effects on T cells, either by modulating apoptosis or the expression of immunosuppressive markers like PD-1 [58, 59]. PGE2 has also been shown to impede IL-2 sensing in T cells, limiting intratumoral generation and the expansion of effector T cell populations [60]. However, the effects of PGE2 on B cells are not known, and to our knowledge, this is the first report of PGE2-induced tBregs that alter T cell responses.

Although immunotherapy has revolutionized the clinical management of cancers in recent years, many patients still do not benefit from this advancement [61]. The majority of the immunotherapy strategies are designed to target the effector T cells. However, it is becoming increasingly obvious that B cells play a significant role in the TME, inducing immunosuppression and influencing immunotherapy responses. Activated B cells can promote the activation and responses of anti-tumoral cytotoxic T cells against tumors [62], implying that B cell activation plays a favorable function in boosting immunotherapy responses [62]. In this regard, the use of B-cell vaccines and anti-CD40 antibody treatment is being investigated [62]. However, targeting pro-tumoral B cells in cancers remains controversial. Initial trials on B cell depletion employing anti-CD20 antibodies have yielded promising results in the treatment of B cell lymphomas, colon cancer, melanoma, and T cell lymphoma [63–66]. However, subsequent studies have revealed that B-cell depletion can have negative consequences in terms of increased tumor growth in mice or eventual relapse in humans [67–70]. This could be attributed to the potential enrichment of low CD20-expressing human and murine Bregs following anti-CD20-mediated B depletion [16]. As a result, the goal would be to target the immunosuppressive TME by precisely suppressing the Bregs while preserving the beneficial B cells. To accomplish this, a better understanding of Bregs, how they form, and their unique markers is required. We believe our study takes a step in that direction by identifying one significant mediator that induces tBregs. Bodogai et al. demonstrated that suppressing CD20^lo^ tBregs in vivo using CXCL13-coupled CpG oligonucleotide (CpG-ODN)-targeted stimulation of B cells prevented cancer metastasis [16]. Pharmacological suppression of the 5-LO/FLAP/PPARα pathway, as well as the usage of resveratrol, has been shown to limit tBreg generation or function, ultimately inhibiting metastasis in the 4T1 tumor [20, 71].

Our study demonstrates that B cell depletion and COX2 inhibitor NS-398 treatment reduced tBreg generation, while TGF-βRI inhibitor SB431542 affected only the suppressive function of tBreg [19]. All three treatments improved T cell responses in vitro and in vivo while reducing tumor growth in tumor-bearing mice. In summary, we have identified anti-CD-19-mediated B cell depletion or COX-2 inhibition as effective immunotherapeutic treatments for eliminating tBregs in soft tissue sarcomas.

While our findings highlight the therapeutic potential of targeting the COX-2/PGE₂-EP2/EP4 axis to disrupt tumor-induced regulatory B cell (Breg) function, it is important to acknowledge the known clinical limitations associated with systemic COX-2 inhibition. A growing number of preclinical and clinical trials have demonstrated the potential effect of COX2/PGE2 blockade in reducing the risk of cancer incidence, progression, and recurrence and improving survival rate in various cancer types. However, whether this treatment strategy would benefit cancer patients remains inconclusive, considering the safety profile issues related to COX2 inhibitors. The most significant concerns associated with COX2 inhibitors involve potential gastrointestinal (GI), cardiovascular (CV), and renal adverse effects [72–76]. Coxibs, like Celecoxib, have demonstrated very low GI adverse effects and negligible renal risk, leaving the major concern as CV risk [77–81]. However, studies have indicated that the increase in CV risk is associated with higher doses and longer duration of the treatment [82, 83]. Despite the potential risks, the incidence of adverse effects associated with COX2 inhibitors at therapeutic doses and durations relevant to cancer treatment is generally manageable.

The main way that COX2 inhibitors raise CV risk is by upsetting the equilibrium between thromboxane A2 (TXA2) and prostaglandin I2 (PGI_2_). COX2 inhibitors decrease PGI2 but leave TXA2 generation unaffected, which might lead to elevated blood pressure, accelerated atherogenesis, and predispose patients receiving Coxibs to an exaggerated thrombotic response to the rupture of an atherosclerotic plaque [72, 84]. These side effects can be overcome by the targeted delivery of COX-2 inhibitors to the tumor microenvironment through nanotherapeutic formulation [85]. Another potential strategy to eliminate this risk is to target EP2/EP4 receptors to maintain the PGI2-TXA2 balance while effectively blocking PGE2 signaling in target cells. EP2/EP4 inhibitors selectively prevent the immunosuppressive effects of PGE2 and may be useful in keeping the immunosuppressive cells, like Breg cells, in the TME under check. This would benefit the anti-tumor immune responses and better cancer management.

However, pharmacologic inhibition of EP2/EP4 or COX-2 can affect multiple immune and stromal cell populations beyond Bregs, raising the possibility of off-target or systemic immunomodulatory effects. These considerations underscore the need for strategies that achieve greater cellular and mechanistic specificity. In this context, targeting tumor-evoked Bregs or their key immunosuppressive mediators, such as TGF-β, may offer a more selective approach to restoring anti-tumor immunity while minimizing widespread immune perturbation. Our data demonstrating that blockade of Breg-derived TGF-β reverses T cell suppression support the functional relevance of this pathway and suggest that Breg-directed interventions could complement or refine upstream COX-2/EP2/EP4 inhibition strategies. Nevertheless, further in vivo studies evaluating therapeutic selectivity, safety, and effects on non-target immune populations will be necessary to determine the translational feasibility of Breg-targeted approaches.

## Experimental section

### Materials

Details of the materials used in this study are summarized in Table S1, supporting information.

### Cell line

The WEHI-164 fibrosarcoma cell line, obtained and authenticated from the National Centre for Cell Sciences (NCCS, Pune, India), was cultured at 37 °C in a humidified environment containing 5% CO_2_ in HiGlutaXL RPMI supplemented with 10% FBS and an anti-bacterial, anti-mycotic solution (Himedia Laboratories, Mumbai, India) (complete medium). No further cell line authentication was done in the laboratory. Experiments were performed using cells that were within 10 passages. The cell line was routinely tested for mycoplasma contamination in-house by DAPI staining.

### Mice

The Institutional Animal Ethics Committee (IAEC), Bhabha Atomic Research Centre, Government of India, authorized the use of animals in this study (BAEC/06/17). BALB/c mice (male) of 6-8 weeks were used for this study. Mice were bred and maintained in the animal house facility of the Bhabha Atomic Research Centre, with food and water *ad libitum*. All experiments were carried out strictly in compliance with the IAEC guidelines for small animal maintenance and dissection.

### Generation of tumor-conditioned medium (TCM)

Tumor-conditioned medium (TCM) was generated from 72 h cultured WEHI-164 cells in a final cell density of 1 × 10^6^ cells/mL. The TCM was filtered through a 0.22 μm sterile syringe filter and stored at –80 °C and used within 30 days. In some experiments, TCM produced from WEHI-164 cells treated with NS-398 (COX-2 inhibitor) (NS-TCM), zileuton (Zil-TCM), or dipyridamole (Dip-TCM) was used.

### Preparation of a single-cell suspension from the spleen

Spleens were pressed through a sterile nylon mesh (70 μM) into RPMI-1640 medium. Red blood cells were lysed with 0.83% ammonium chloride solution. The resultant lymphocyte cell suspension was washed with RPMI-1640 medium and resuspended in complete medium and used in all experiments.

### Purification of CD19^+^ B cells and B-depleted T cells

Spleen cells were treated with anti-mouse CD19-coated magnetic microbeads according to the manufacturer's instructions and purified using an LS column in VarioMACS™ (Miltenyi Biotec, Gladbach, Germany). The cells eluted from the column were tested for purity using flow cytometry. The flow-through cells were used as B-depleted T cells (B_dep_). The purity of the population used in the study was consistently greater than 95%.

For purification of TGF-β^+^ Bregs, Bregs were incubated with rat anti-mouse LAP-TGFβ1 antibody, followed by treatment with anti-rat IgG magnetic microbeads. The labeled cells were purified using an LS column, and purity was checked using flow cytometry. The flow through cells was used as TGF-β^-^Bregs.

### Generation of Bregs

Bregs were produced by treating CD19^+^ B cells (1 × 10^6^ cells/mL) with TCM (20%), NS-TCM (20%), or PGE2 (10 µM) for 72 h at 37 °C in a humid incubator with 5% CO_2._ Untreated B cells, cultured in complete medium, served as the control (B-UT). After treatment, the cells were collected, washed in fresh complete medium, counted with trypan blue, and used for further experiments. For inhibition of signaling pathways, B cells were incubated with corresponding inhibitors for 2 h, followed by the Breg generation protocol.

For siRNA-mediated depletion of proteins in Bregs, a pool of three in vivo modified siRNAs targeting the ORF of the gene of interest was used. Purified B cells were resuspended in siRNA delivery medium and seeded at 1 × 10^6^ cells/well/500 μL in 24-well plates. SiRNAs were directly added to the culture at a 1 μM final concentration and incubated for 6 h at 37 °C in a humid incubator with 5% CO_2_. The culture was then supplemented with 10% FBS and incubated overnight. The next day, cells were harvested, re-suspended in complete medium, and an equal number of viable cells were cultured with TCM for the generation of Breg cells.

### Breg-T cell suppression assay

CFSE (carboxyfluorescein succinimidyl ester)-labeled B_dep_ cells (1 × 10^6^ cells/mL) were co-cultured with Breg cells (following different treatments) in a 1:1 ratio. These cells were stimulated with anti-CD3/CD28 magnetic beads (Dynabeads™ Mouse T-Activator CD3/CD28, Gibco) at a 3:1 ratio and incubated for 72 h at 37 °C in a humidified incubator with 5% CO_2_. After 72 h, the beads were removed with a magnet, and the cells were harvested and labeled with fluorochrome-conjugated anti-CD4 or anti-CD8 antibodies. The proliferation of gated cells was measured by flow cytometry. The gating strategy used for the CFSE dilution assay in B_dep_ T cells is given in Fig. S10A, supporting information.

### Breg-Treg generation assay

MACS-purified CFSE-labeled naïve CD4^+^ T cells were co-cultured with Breg cells at a 1:1 ratio. They were then stimulated with anti-CD3/CD28 magnetic beads at a 2:1 ratio for 72 h at 37 °C in a humidified incubator with 5% CO_2_. After incubation, the beads were removed, and CD4^+^CD25^+^Foxp3^+^ Tregs were examined by flow cytometry (Cyflowspace™ flow cytometer (Sysmex, Germany) or Spectral: NovoCyte Opteon Agilent Technologies, CA, USA). The gating strategy used for Treg analysis is given in Fig. S11A, B, supporting information.

### Cytotoxicity assay

For Breg-mediated cytotoxicity in T cells, purified B_dep_ T cells were stained with 5 μM calcein-AM and co-cultured with B cells. After incubation for 24 h, culture supernatants were transferred into a new 96-well plate, and fluorescence was measured using a spectrofluorometer (Biotek Synergy H1, excitation 485 nm and emission 525 nm).

T cell cytotoxicity was assessed by the target WEHI-164 cell killing assay. T cells purified from the B: T co-culture assay were cultured for 24 h with calcein-AM-stained WEHI-164 cells; the supernatants were removed and fluorescence measured. Fluorescence from target cells only served as a negative control (spontaneous release), and target cells treated with 2% Triton-X 100 served as a positive control (maximum release). The percentage of lysis was calculated as % cytotoxicity = 100 x (test well-spontaneous release)/ (maximum release-spontaneous release).

### Flow cytometry analysis

Single-cell suspensions were incubated with 10% FBS in PBS for 20 min to prevent nonspecific antibody binding. The cells were then treated with fluorochrome-conjugated antibodies for 30 min at room temperature in the dark. For intracellular staining, cells were fixed with 2% formaldehyde solution and permeabilized in 0.3% Tween-20 and 0.5% FBS in PBS. For Foxp3 staining, 0.3% Triton X-100 was utilized for permeabilization. Cells were incubated for 30 min with fluorochrome-conjugated antibodies before being washed and resuspended in PBS. Appropriate isotype controls were included. Samples were acquired using the Cyflowspace™ flow cytometer (Sysmex, Germany) with Flowmax software. Data analysis was performed with either FCS Express or FlowJo software.

### Western blotting

Breg cells generated under various conditions were subjected to SDS-PAGE and Western blotting for analysis of protein expression. In brief, cell lysates were produced from an equal number of cells using 1X sample loading buffer that contained protease and phosphatase inhibitors. Equal amounts of protein samples were electrophoresed in a 10% SDS-polyacrylamide gel. The proteins were transferred to a PVDF membrane, which was blocked for 1 h with 4% bovine serum albumin (BSA) in TBS containing 0.05% Tween-20 (TBST) and then incubated overnight with appropriate primary antibodies with gentle agitation. To eliminate any unbound primary antibodies, the blots were carefully washed three times with TBST before being treated for 2 h with horseradish peroxidase-conjugated secondary antibody. The blots were again washed with TBST, and bands were developed using an enhanced chemiluminescence detection kit (Immobilon Western chemiluminescent HRP substrate, Millipore, MA, USA) in a gel documentation system (Syngene, Cambridge, UK). The band intensities were measured with ImageJ software. The original uncut version of the blots used in this article is given in supporting file S2.

### In vivo B cell depletion

Three days before the tumor challenge, BALB/c mice were depleted of B lymphocytes using InVivoMAb anti-mouse CD19 (Bioxcell). Mice were injected intraperitoneally with anti-CD19 antibody (250 μg/mouse). B-cell depletion was confirmed on days 3 and 20 using flow cytometry.

### Tumor generation and treatments

WEHI-164 cells (2 × 10^5^) were injected intramuscularly into the right flanks of BALB/c mice. Palpable tumors were visible on day 8. Tumor volume was assessed using digital Vernier calipers, beginning on the day the tumor became palpable. Tumor volume was computed using the ellipsoid volume formula with the assumption that the tumor’s height equals its width [86]. All tumor-bearing mice (TBM) were sacrificed on day 20 and used for experiments. Non-tumor (NT) mice served as controls.

For B-depletion studies, the tumor was inoculated 3 days after in vivo B-depletion. For B cell adoptive transfer experiments, Bdep TBM were randomized into three groups, and B cells (20 × 10^6^) from NT mice (Bdep TBM + B-NT) or WEHI-164 tumor-bearing mice (Bdep TBM + Breg) were administered by i.v. route. Untreated Bdep-TBM served as controls. Adoptive transfer was performed on days 3, 10, and 17 after tumor challenge to ensure the continuous presence of B cells. For NS-398 studies, tumor-inoculated mice were administered i.p. with either 50 μL dimethyl sulfoxide (DMSO-vehicle) or NS-398 (10 mg/kg body weight) for 10 days.

### Bioinformatics analysis

#### Gene expression profile datasets and survival curves

Gene-level expression (batch-normalized TPM, log_2_(normalized value + 1), and clinical survival data for TCGA-Pancancer cohorts were downloaded from UCSC Xena. Sample-level data included overall survival time, survival status, and expression of genes under study. Samples with missing survival status, time, or CD79A/PTGS2/TGFB1 expression were excluded. Samples with CD79A expression ≥2.58 were filtered in as samples with strong B cell marker association. They were then stratified into “PTGS2-high” and “PTGS2-low” using the survival cutoff point determined by *survminer::surv_cutpoint*. TGFB1 expression was stratified into quartiles; ≤Q1 was treated as “Normal” and ≥Q3 as “Breg.” These samples were further annotated into “Low-Normal,” “High-Normal,” “Low-Breg,” and “High-Breg.” Kaplan-Meier curves were fit with *survival::survfit* and plotted via *survival::ggsurvplot*. Cox proportional hazard models (surv::coxph) were used to estimate the hazard ratio with 95% confidence intervals. Log_2_TPM values for genes of interest were normalized with z-scoring and scaled to a common range, followed by heatmap generation using the *pheatmap* function. All analyses were done in R version 4.5.1.

#### Single-cell RNA-seq datasets and analysis

The raw files of single-cell RNA sequencing (scRNA-seq) datasets for osteosarcoma (GSE162454), other sarcoma subtypes (GSE279852), breast cancer, colorectal cancer, and ovarian cancer (GSE242271) were downloaded from Gene Expression Omnibus (GEO). Raw gene-cell count matrices were processed using the Seurat package in R. For each sample, a Seurat object was created, and after quality control, they were log-normalized, integrated using *RunHarmony*, and PCA was calculated using *RunPCA()*. Dimensionality reduction and primary cell clustering were performed on the data using the *RunUMAP*. Clusters were annotated based on canonical marker expression using *WhichCells*. B cells were annotated by the expression of *CD79A*, *MS4A1*, and *CD19*; NK/T cells by *CD2*, *CD3D*, *CD3E*, *CD3G*, *NKG7*, *KLRD1*, and *KLRB1*; cancer-associated fibroblasts by *FAP*, *VIM*, *COL1A1*, *COL1A2*, and *PDGFRA*; endothelial cells by *EGFL7* and *PLVAP;* and myeloid cells by *CD14*, *CD68*, *LYZ*, *FCGR3A*, and *PPBP*. B cells were classified into different subsets and reprocessed independently, including normalization, PCA, UMAP, and clustering. Expression of *TGFB1* across the B cell clusters was examined by violin and feature plots. Clusters with high *TGFB1* expression were designated as “TGF-β^hi^,” and clusters with low or absent expression were designated as “TGF-β^lo^.” Differentially expressed genes (DEGs) were identified between the groups, keeping the TGF-βl^o^ group as a reference using *FindMarkers*. All the above-mentioned analyses were executed using the functions available in the Seurat v4 package [87]. The pathway analysis was carried out on the DEGs using the function gseGO from the R package *clusterProfiler* [88]. The differential genes and pathways were plotted using the heatmap function with *ComplexHeatmap*. All scRNA-seq bioinformatic analysis was performed on R version 4.3.2.

### Statistical analysis

All experiments were repeated at least three times. The number of repetitions is mentioned in the figure legends. Each data point represents one biological replicate. Sample sizes were chosen to yield statistical significance based on previous studies and literature. No statistical studies were conducted to determine sample sizes. Investigators were not blinded during the allocation or analysis of in vitro and ex vivo experiments due to the objective nature of flow cytometry and Western blotting quantifications. For tumor measurement experiments, tumor-inoculated mice were randomly assigned to treatment groups prior to treatment initiation to ensure similar tumor distribution. For NS-398 administration experiments, the injection was performed single-blinded. No blinding was done for B depletion or adoptive transfer experiments. Mice that did not develop visible tumors were excluded from the study.

All statistical analyses were performed using GraphPad Prism. Data are reported as mean ± SEM. Aside from basic statistics (mean, standard deviation, and standard error of the mean), Student’s t-test and ANOVA were used where appropriate. Normality and log normality of both in vitro and in vivo data were checked using the Shapiro–Wilk test. The significance between the two independent variables was determined using the Student’s two-tailed equal variance t-test. To compare more than two variables, one-way ANOVA was used. No adjustments for multiple comparisons were applied to the in vitro data. For tumor measurement data comparing more than two groups, two-way ANOVA (mixed-effect model (REML)) was used. Multiple comparisons were adjusted using Dunnett’s test. The results and the figure legends both report significance levels. Figures indicate significance with symbols denoting *p ≤ 0.05, **p ≤ 0.01, ***p ≤ 0.001, and ****p ≤ 0.0001.

## Supplementary information

Supporting file S2- Uncropped western blots

Supplementary Table and Figures

## Data Availability

The corresponding author can provide the data that support the findings of this study upon reasonable request.

## Code availability

The custom codes generated for this study are available from the corresponding author upon reasonable request.
