# Supplementary material for: TGF-β-producing tBregs are associated with poor survival rates in PTGS2-high cancers
Source: Cell Death Dis. 2026 Jun 26;17(1):795. doi: 10.1038/s41419-026-09045-w (PMC13572553; doi:10.1038/s41419-026-09045-w)
Supplement: Supplementary file 1 [file 41419_2026_9045_MOESM1_ESM.pdf]

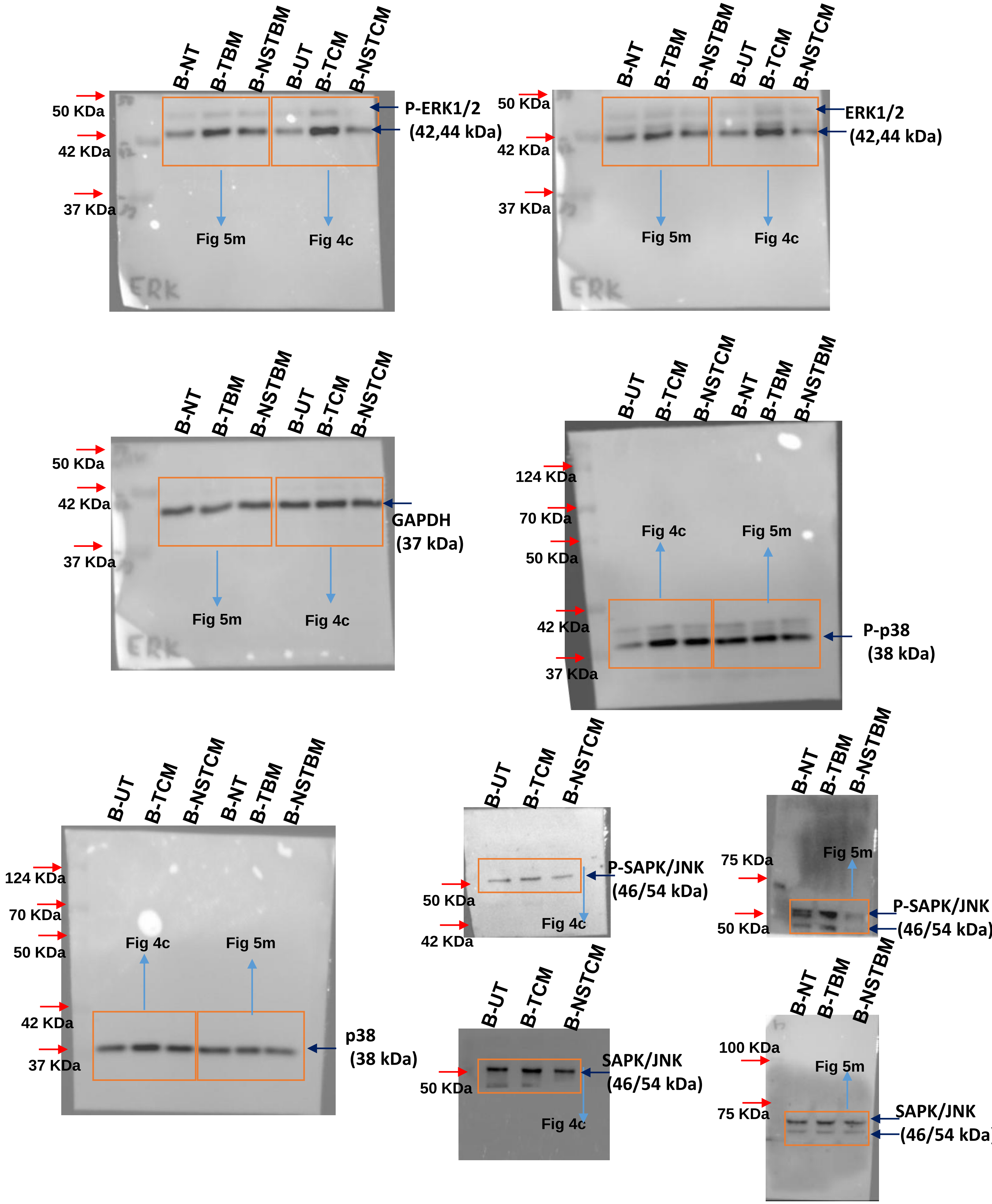
B-NT
B-TBM
B-NSTBM
B-UT
B-TCM
B-NSTCM
50 KDa
42 KDa
37 KDa
P-ERK1/2
(42,44 kDa)
Fig 5m
Fig 4c
ERK
ERK1/2
(42,44 kDa)
GAPDH
(37 kDa)
124 KDa
70 KDa
P-p38
(38 kDa)
p38
(38 kDa)
P-SAPK/JNK
(46/54 kDa)
75 KDa
SAPK/JNK
100 KDa

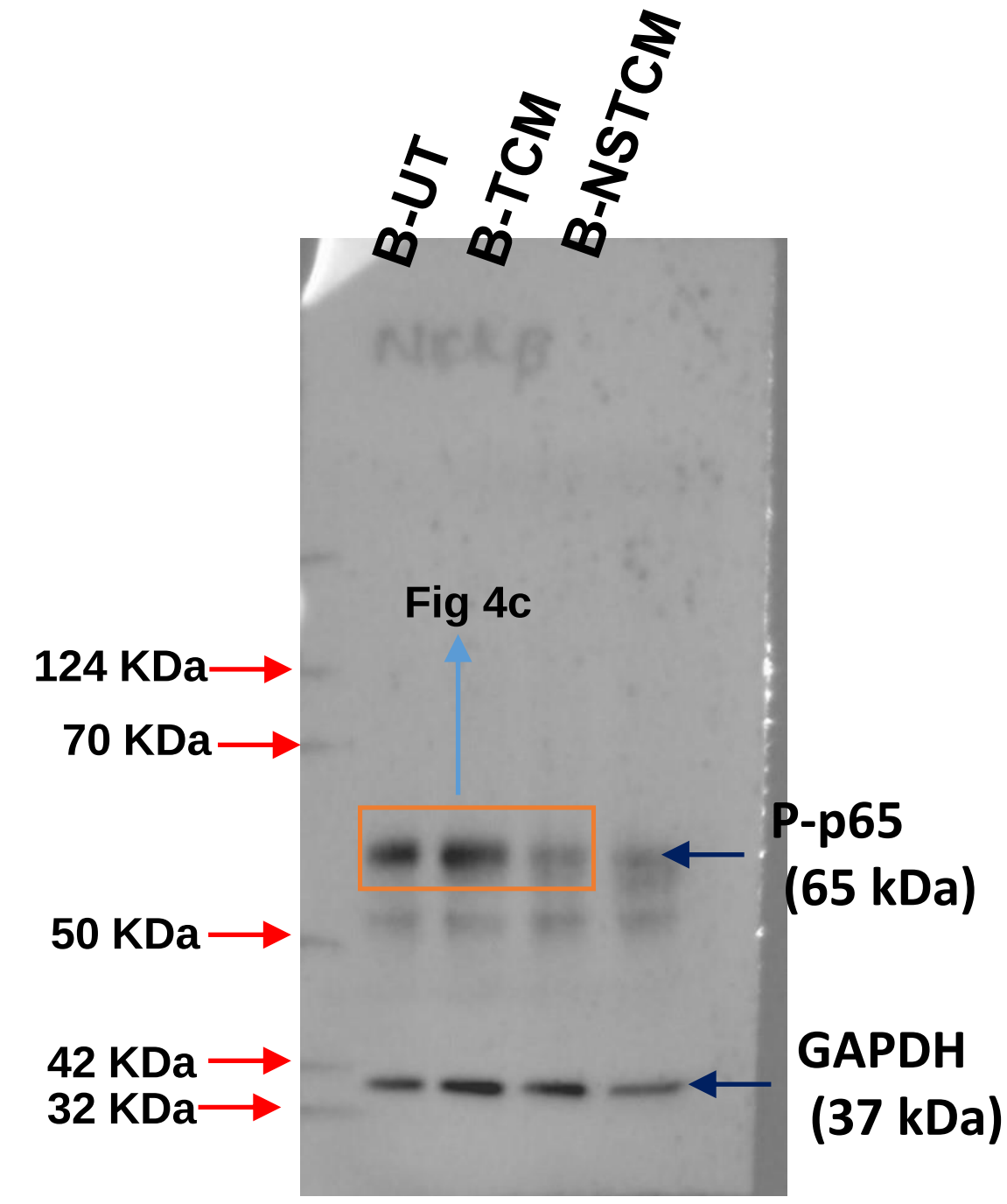
B-UT
B-TCM
B-NSTCM
Fig 4c
124 KDa
70 KDa
50 KDa
42 KDa
32 KDa
P-p65
(65 kDa)
GAPDH
(37 kDa)

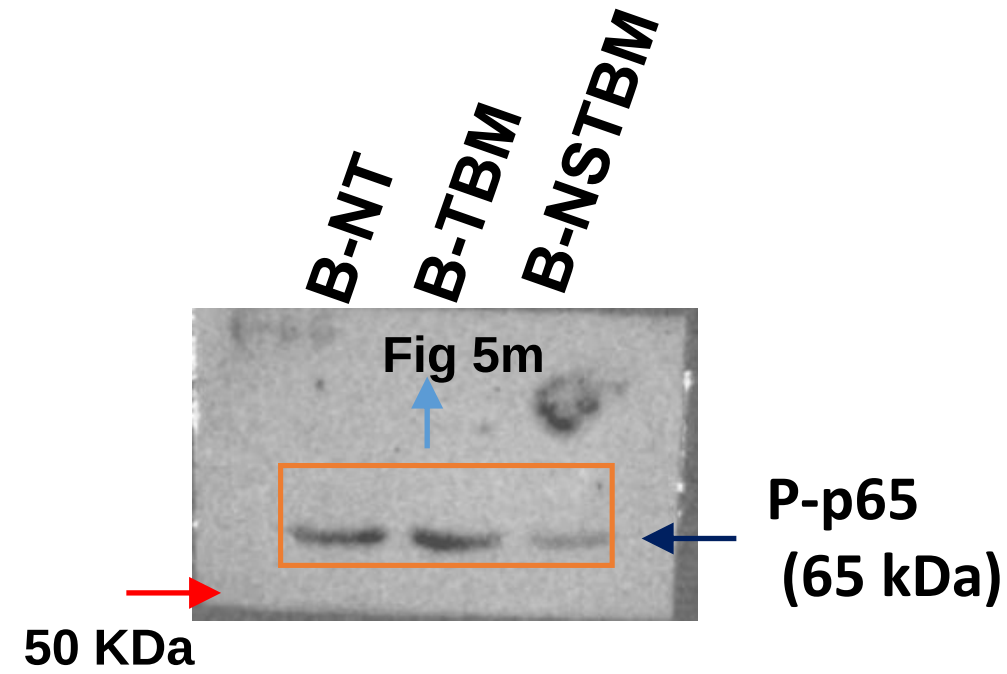
B-NT
B-TBM
B-NSTBM
Fig 5m
50 KDa
P-p65
(65 kDa)

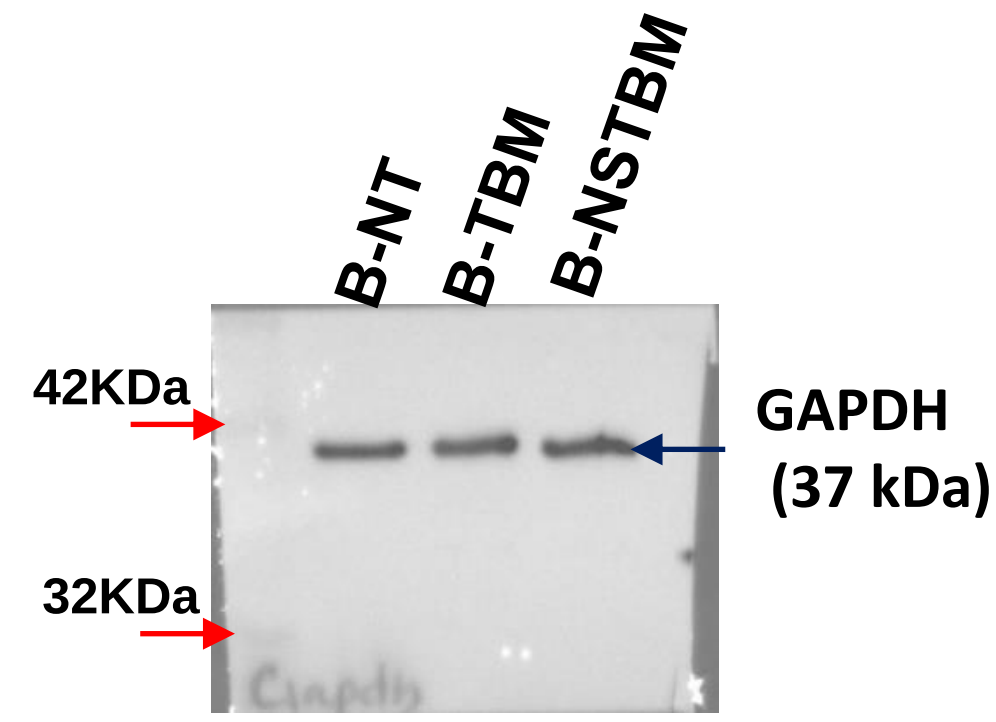
B-NT
B-TBM
B-NSTBM
42KDa
32KDa
GAPDH
(37 kDa)

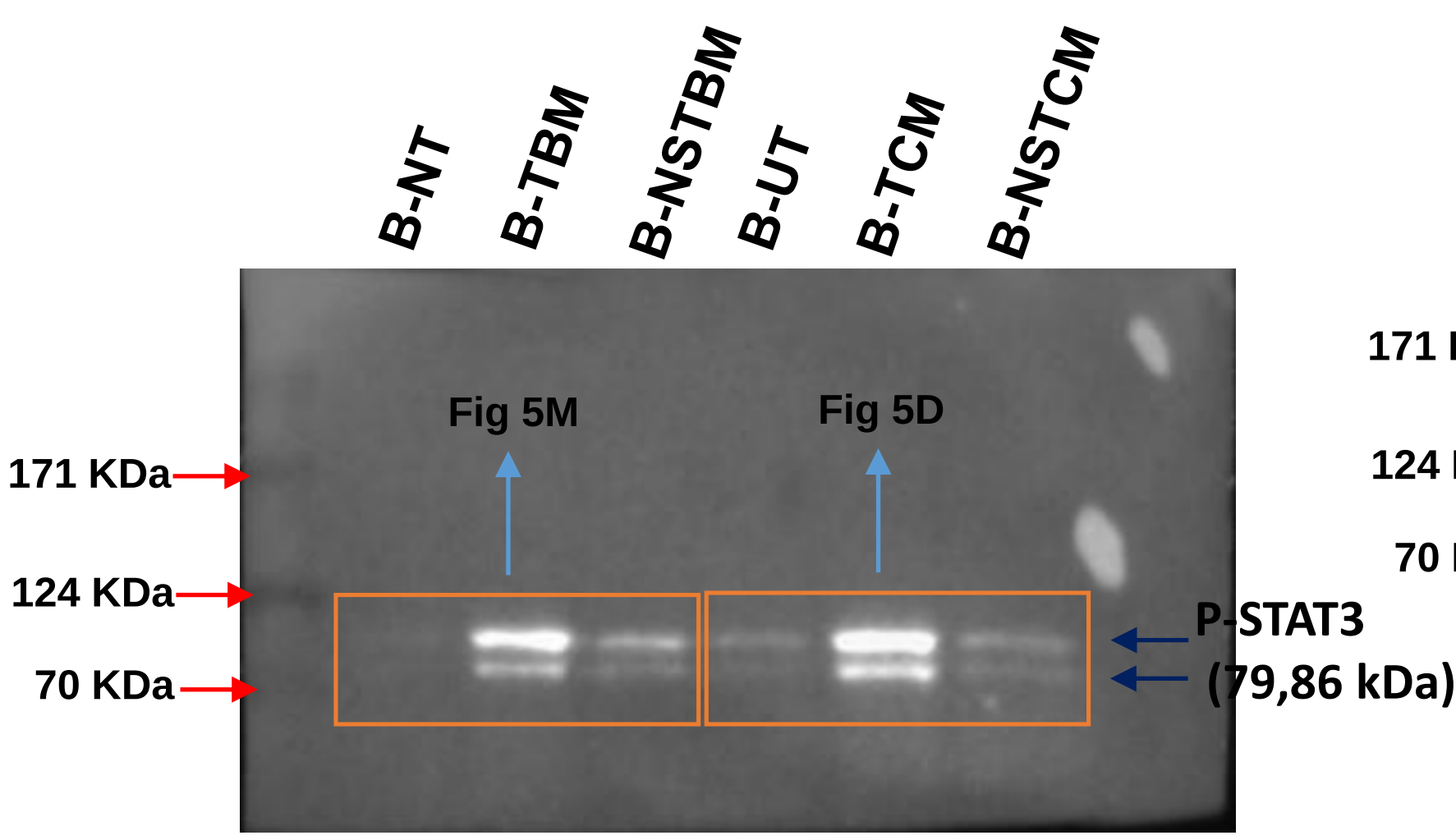
B-NT
B-TBM
B-NSTBM
B-UT
B-TCM
B-NSTCM
Fig 5M
Fig 5D
171 KDa
124 KDa
70 KDa
P-STAT3
(79,86 kDa)

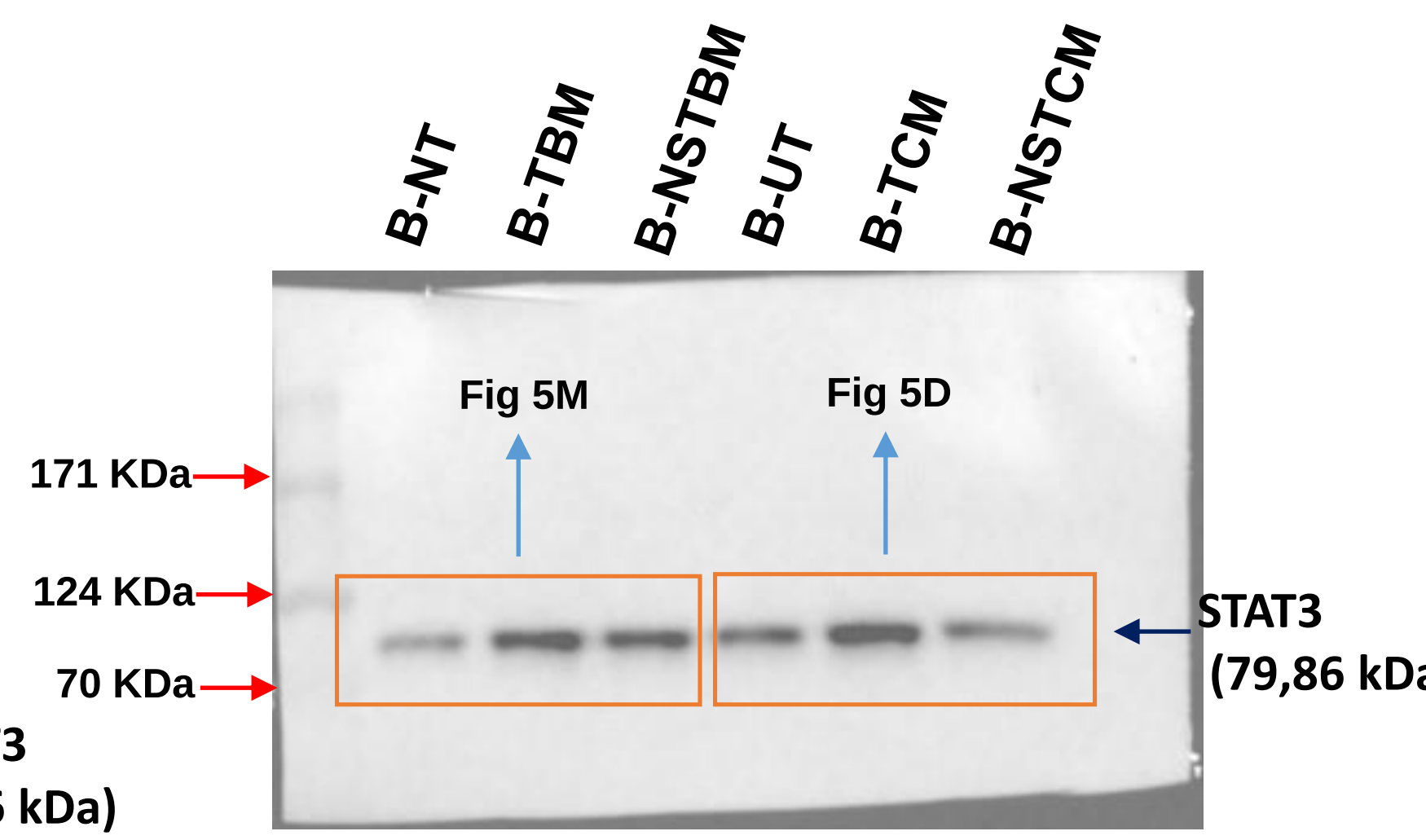
B-NT
B-TBM
B-NSTBM
B-UT
B-TCM
B-NSTCM
Fig 5M
Fig 5D
171 KDa
124 KDa
70 KDa
STAT3

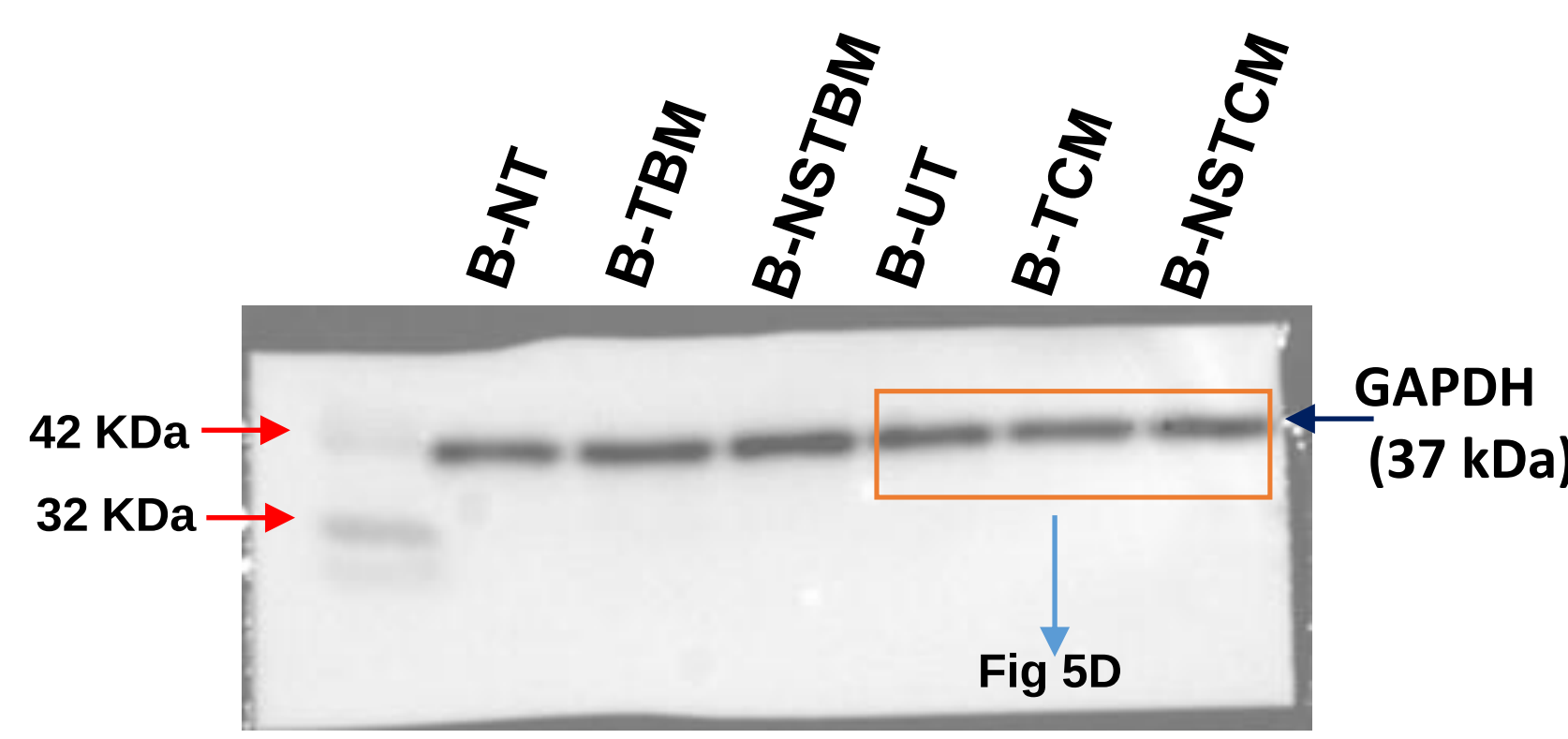
B-NT
B-TBM
B-NSTBM
B-UT
B-TCM
B-NSTCM
42 KDa
32 KDa
GAPDH
Fig 5D

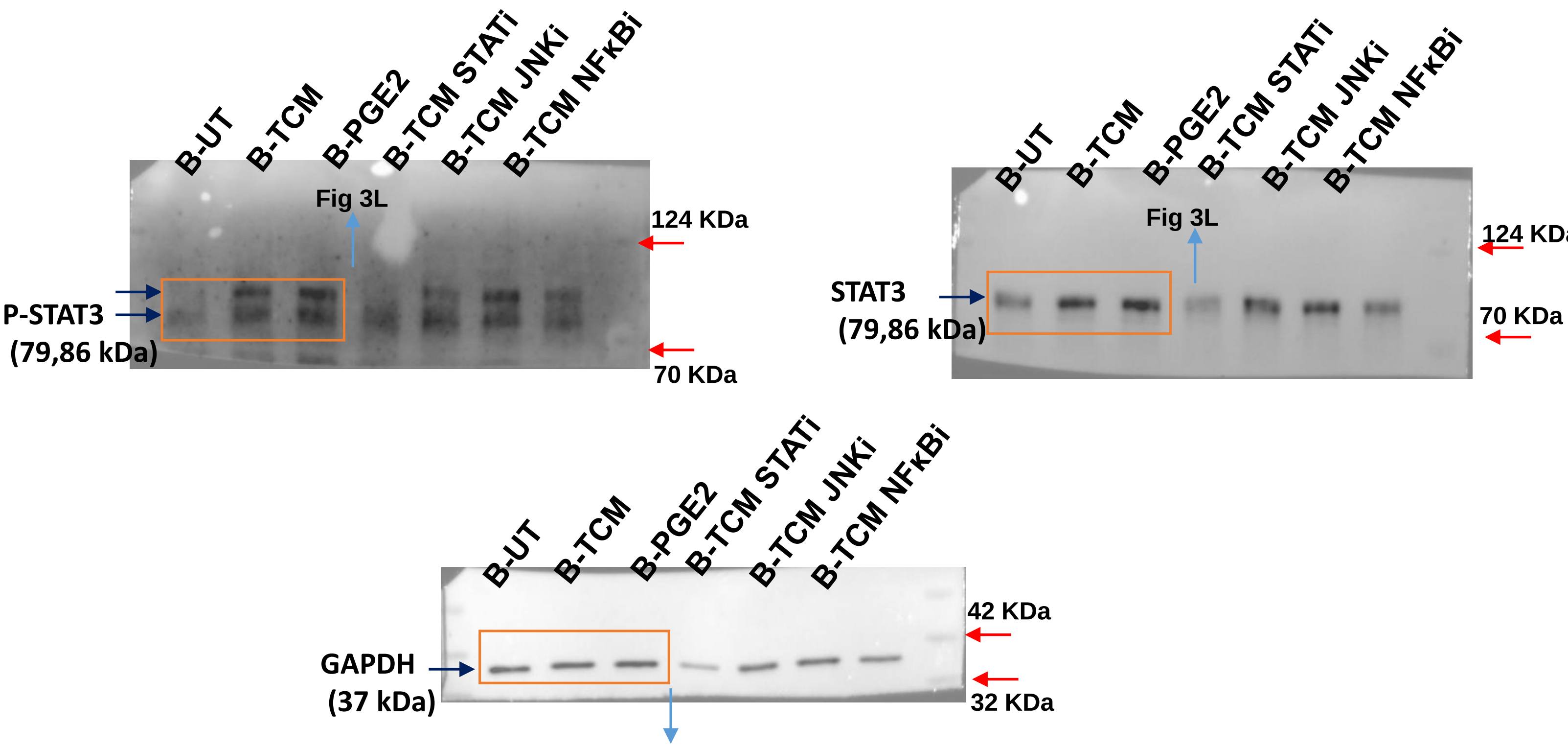
B-UT
B-TCM
B-PGE2
B-TCM STATi
B-TCM JNKi
B-TCM NFκBi
Fig 3L
124 KDa
70 KDa
P-STAT3
(79,86 kDa)
STAT3
(79,86 kDa)
GAPDH
(37 kDa)
42 KDa
32 KDa

Fig 3L

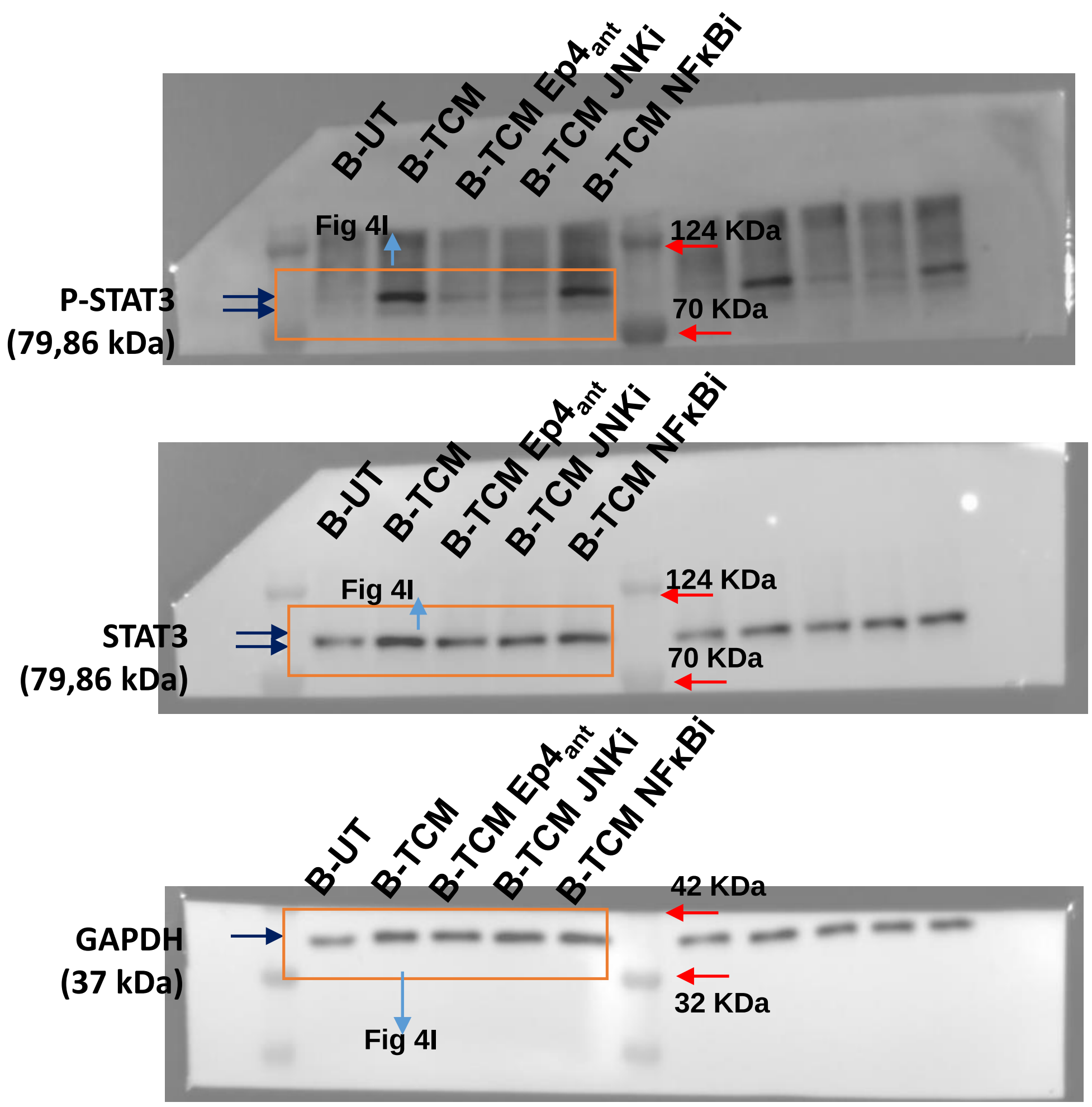
B-UT
B-TCM
B-TCM Ep4ant
B-TCM JNKi
B-TCM NFκBi
Fig 4I
124 KDa
70 KDa
P-STAT3
(79,86 kDa)
STAT3
(79,86 kDa)
GAPDH
(37 kDa)
42 KDa
32 KDa

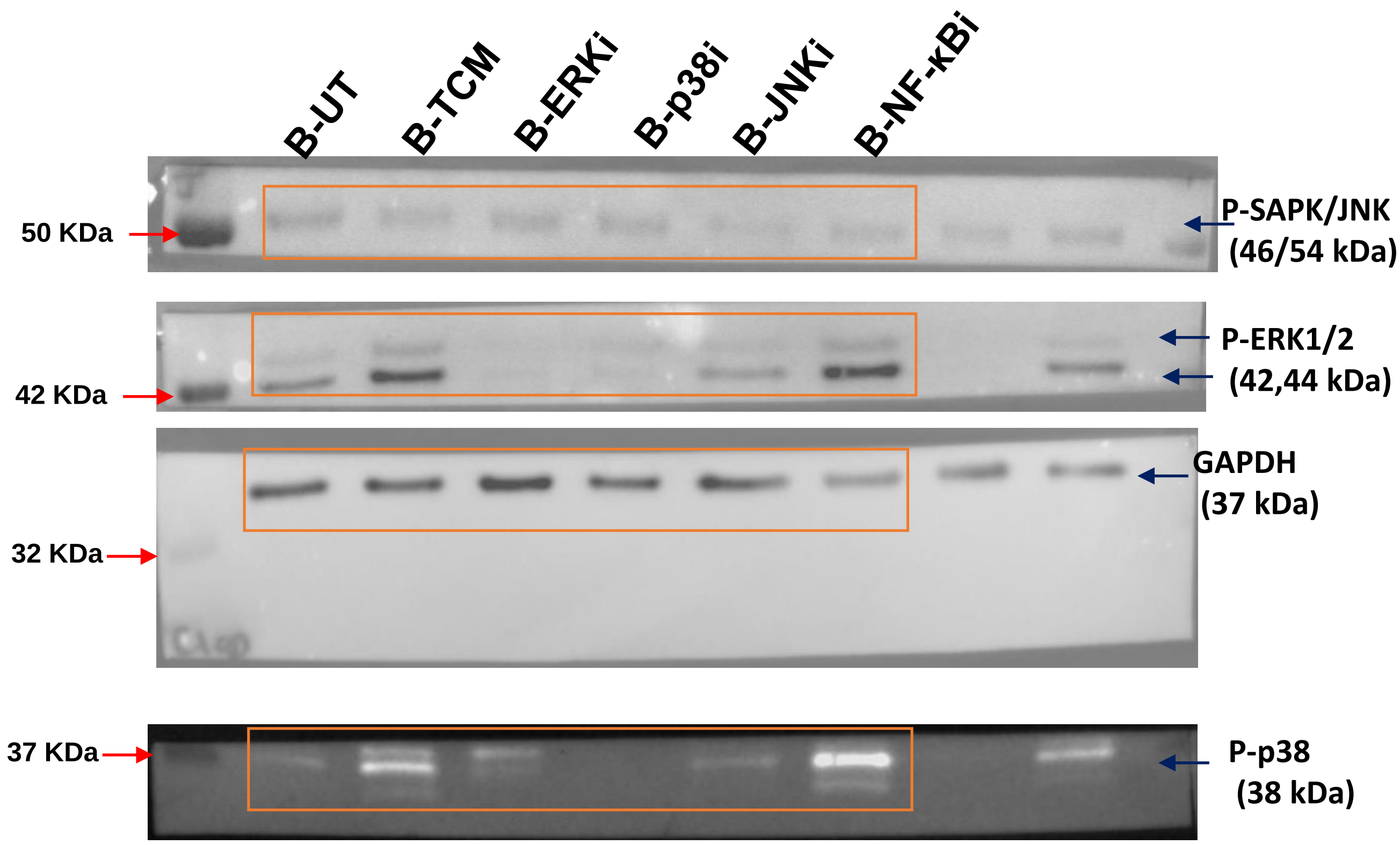

Supplementary figure S4A
