# Supplementary material for: TGF-β-producing tBregs are associated with poor survival rates in PTGS2-high cancers
Source: Cell Death Dis. 2026 Jun 26;17(1):795. doi: 10.1038/s41419-026-09045-w (PMC13572553; doi:10.1038/s41419-026-09045-w)

Supporting Information

TGF-β producing tBregs are associated with poor survival rates in PTGS2-high cancers

Kavitha Premkumar*[1], *Soundharya Ramu*[2], *Mohit Kumar Jolly*[3] *and Bhavani S. Shankar*[1,4]**

[1]Immunology Section, Radiation Biology & Health Sciences Division, Bio-Science Group,
Bhabha Atomic Research Centre, Mumbai-400 085, India

[2]Interdisciplinary Mathematics Initiative, Indian Institute of Science, Bengaluru 560012, India

[3]Department of Bioengineering, Indian Institute of Science, Bengaluru 560012, India

[4]Homi Bhabha National Institute, Anushaktinagar, Mumbai 400 094, India

* Corresponding author.

Dr. Bhavani S. Shankar (bshankar@barc.gov.in)

Table S1: Antibodies and Reagents

List of antibodies used in this study		
Antibody	Manufacturer	Catalogue Number
Flow cytometry		
CD19,PE (1D3)	BD Biosciences, CA, USA	557399
CD19, PerCP Cy5.5 (1D3)		551001
CD3, PE (17A2)		555275
CD4, PE (GK1.5)		557308
CD4, PerCP Cy5.5(RM4-5)		550954
CD8, PerCP Cy5.5 (53-6.7)		551162
CD25, PE (PC 61)		553866
Foxp3, Alexa Fluor488 (MF23)		560403
CD69, FITC (H1.2F3)		557392
CD27,PE (LG.3A10)		558754
CD81, PE (Eat2)		559519
CD86, FITC (GL1)		561962
CD40, FITC (3/23)		561845
CD80, FITC (16-10A1)		561954
IA/IE, FITC (2G9)		553623
Rat IgG2b, κ Isotype Control, PE (A95-1)		553989
Rat IgG2a, κ Isotype Control, PerCP Cy5.5 (R35-95)		550765
Rat IgG2a, κ Isotype Control, PE (R35-95)		553930
Rat IgG2b, κ Isotype Control, Alexa Fluor488 (A95-1)		557726
Hamster IgG2 κ Isotype Control, FITC (B81-3)		550056
Rat IgG1, λ Isotype Control, PerCP Cy5.5 (A110-1)		551072
Hamster IgG1 κ Isotype Control, PE (A19-3)		553972
Hamster IgG1, λ1 Isotype Control, FITC (G235-2356)		553953
Rat IgG2a, κ Isotype Control, FITC (R35-95)		553929
PDL1,PE (MIH7)	Biolegend, San Diego, CA	155402
LAP-TGF-β1, FITC (TW7-16B4)		141414
Mouse IgG1, κ Isotype Control, FITC (MOPC-21)		400108
EP2 (H-75)	Santa Cruz Biotechnology, Santa Cruz, CA	SC-20675
EP4 (C4)		SC-55596
anti-mouse IgG, Alexa fluor 488 (goat polyclonal)	Thermo Fisher Scientific, MA, USA	A11001
anti-rat IgG, Alexa fluor 488 (donkey polyclonal)		A21208
REAfinity CTLA-4 PE (REA984|UC10-4B9)	MilteyiBiotec, Gladbach, Germany	130-116-454
REAfinity TIM-3 PE (REA602|RMT3-23)		130-118-691
REAfinity PD-1 PE (REA802|HA2-7B1)		130-111-800
REAfinity LAG-3 PE (REA776|C9B7W)		130-111-327
REAfinity TIGIT-PE (REA536|GIGD7)		130-120-296
REAfinity CD4 PerCP Vio 700 (REA604|RM4-5)		130-105-876
REAfinity CD8a PerCP Vio 700 (REA601|53-6.7)		130-128-616
REAfinity Granzyme B FITC (REA226|GB11)		130-118-480
REAfinity CD25 VioBright FITC (REA568|7D4)		130-120-088
REAfinity Foxp3 PE (REA788|3G3)		130-111-603

Western blotting		
P-STAT3 (Tyr705) (M9C6)	Cell Signaling Technology, MA, USA	4113
STAT3 (79D7)		4904
P-NFκB p65 (Ser536) (93H1)		3033
NFκB p65 (D14E12)		8242
P-SAPK/JNK (Thr183/Tyr185) (81E11)		4668
SAPK/JNK	Cell Signaling Technology, MA, USA	9252
P-p44/42 (Thr202/Tyr204) (D.13.14.4E)		4370
p44/42 (137F5)		4695
P-p38 (Thr180/Tyr182) (D3F9)		4511
p38 (D13E1)		8690
GAPDH (14C10)		2118
Anti-rabbit IgG, HRP-linked		7074
Anti-mouse IgG, HRP-linked		7076

Magnetic microbeads		
CD19 microbeads, mouse	MilteyiBiotec, Gladbach, Germany	130-121-301
Anti-Rat IgG microbeads		130-048-502
Dynabeads Mouse T-Activator CD3/CD28	Gibco, Thermo Fisher Scientific, MA, USA	11453
In vivo antibody		
InVivoMAb anti-mouse CD19	BioXcell, NH, USA	BE0150

List of other reagents used in this study

Item	Manufacturer	Catalogue number
SiRNAs		
Mouse Ptger4 (19219) siRNA-SMARTpool (pool of 3 ORF targets)	Dharmacon Inc., CO, USA	E-048700-00-0010
Mouse Stat3(20848) siRNA-SMARTpool (pool of 3 NTC)	Invitrogen, Thermo Fisher Scientific, MA, USA	E-040794-00-0010
Mouse Non-target control SiRNA (20848) siRNA-SMARTpool (pool of 3 ORF targets)	R7D systems (MN,USA)	D-001910-10-05
Accell siRNA Delivery Media		B-005000-500
ELISA kits		
Mouse IL-6	BD Biosciences, CA, USA	555240
Mouse IL-10		555252
Human/mouse TGF-β1	Invitrogen, Thermo Fisher Scientific, MA, USA	50-174-90
Prostaglandin E2 parameter assay kit	R7D systems (MN,USA)	KGE004
Inhibitors and Reagents		
HiglutaXL RPMI	Himedia Laboratories, Mumbai, India	AL060G
Fetal bovine serum		AL060G
Anti-bacterial anti-mycotic solution		AL060G
DMSO	Sigma Aldrich,MO, USA	AL060G
NS-398		N194
PGE2		P0409
PF-04418948		PZ0213
MK2894		
PF-04217329		PZ0369
IL-6 mouse, recombinant		SRP3330

CY10598	Cayman Chemical, MI,USA	13281
CellTrace CFSE Cell Proliferation Kit	Invitrogen, Thermo Fisher Scientific, MA, USA	C34554
Calcein AM		C34852
BP-1-102	MedChemExpress, NJ, USA	HY-100493
UO126	Abcam, Cambridge, UK	ab120241
SB203580		ab120162
SP600125		ab120065
Zileuton sodium	Targetmol, MA, USA	T21836
Dipyridamole		T1618
NFKB activation inhibitor III	Millipore, MA, USA	481411
Immobilon western chemiluminescent HRP substrate		WBKLS0500

Figure S1: TGF-β expressing Breg cells in sarcoma

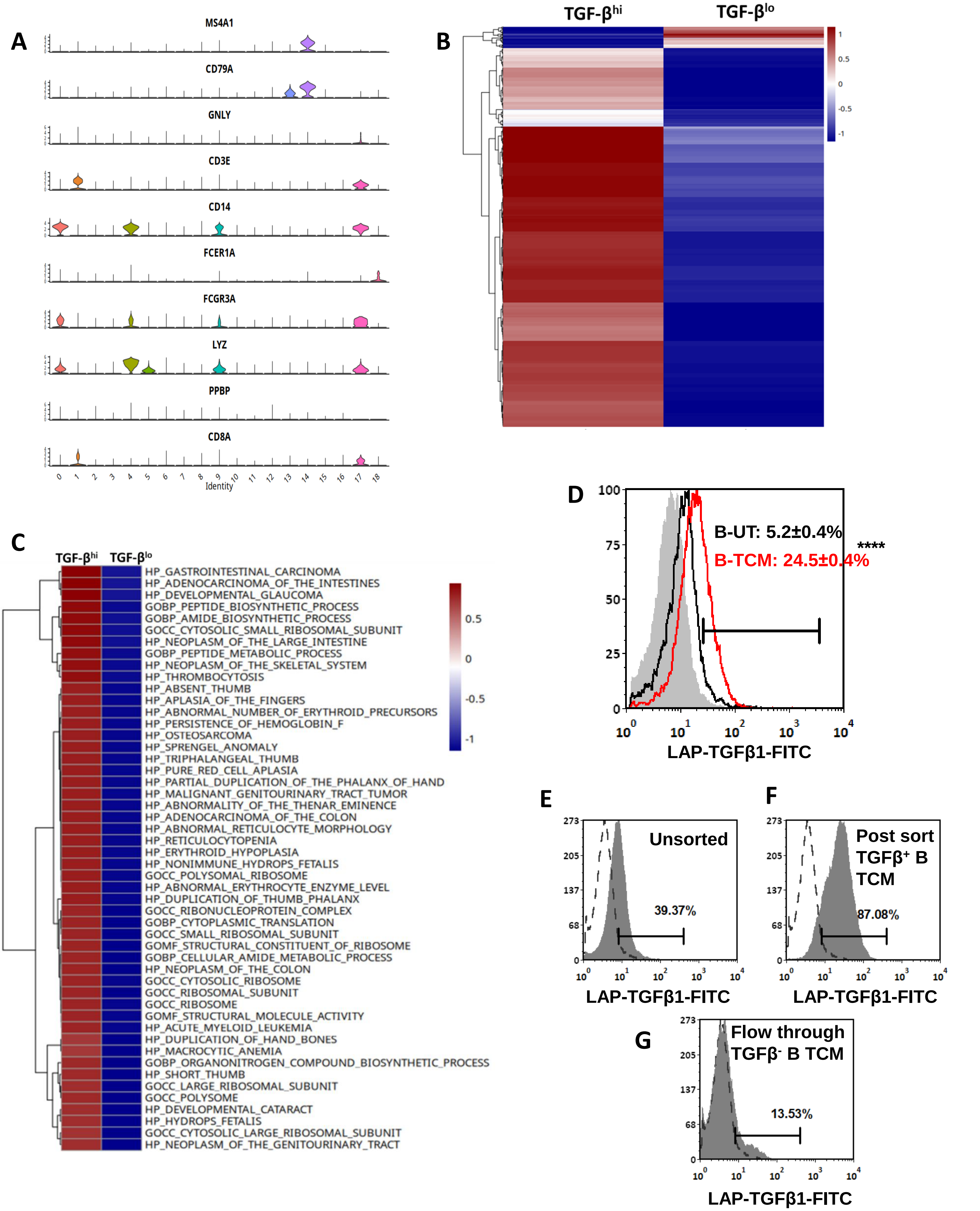

Figure S1: TGF-β expressing Breg cells in sarcoma. (A) Feature plot showing canonical cell annotation markers, with clusters color coded same as the UMAP projection. (B) Heatmap showing relative (z-scored per row) expression of differentially regulated genes in TGF-β^{hi} and TGF-β^{lo} B cells. (C) Heatmap showing the normalized expression scores of the top 50 upregulated pathways in the TGF-β^{hi} B cells. (D) Expression of surface membrane bound TGFβ in B cells cultured with TCM (B-TCM). TGF-β^{+} and TGF-β^{-} cells were sorted from B-TCM cells using anti-LAP-TGF-β1 antibody followed by MACS using anti-rat IgG magnetic beads. Surface TGF-β expression in (E) un-sorted B-TCM cells, (F) purified TGF- β^{+} cells (eluant) and (G) TGF- β^{-} cells (flow through)

Figure S2: B cell depletion in WEHI-164 tumor bearing mice

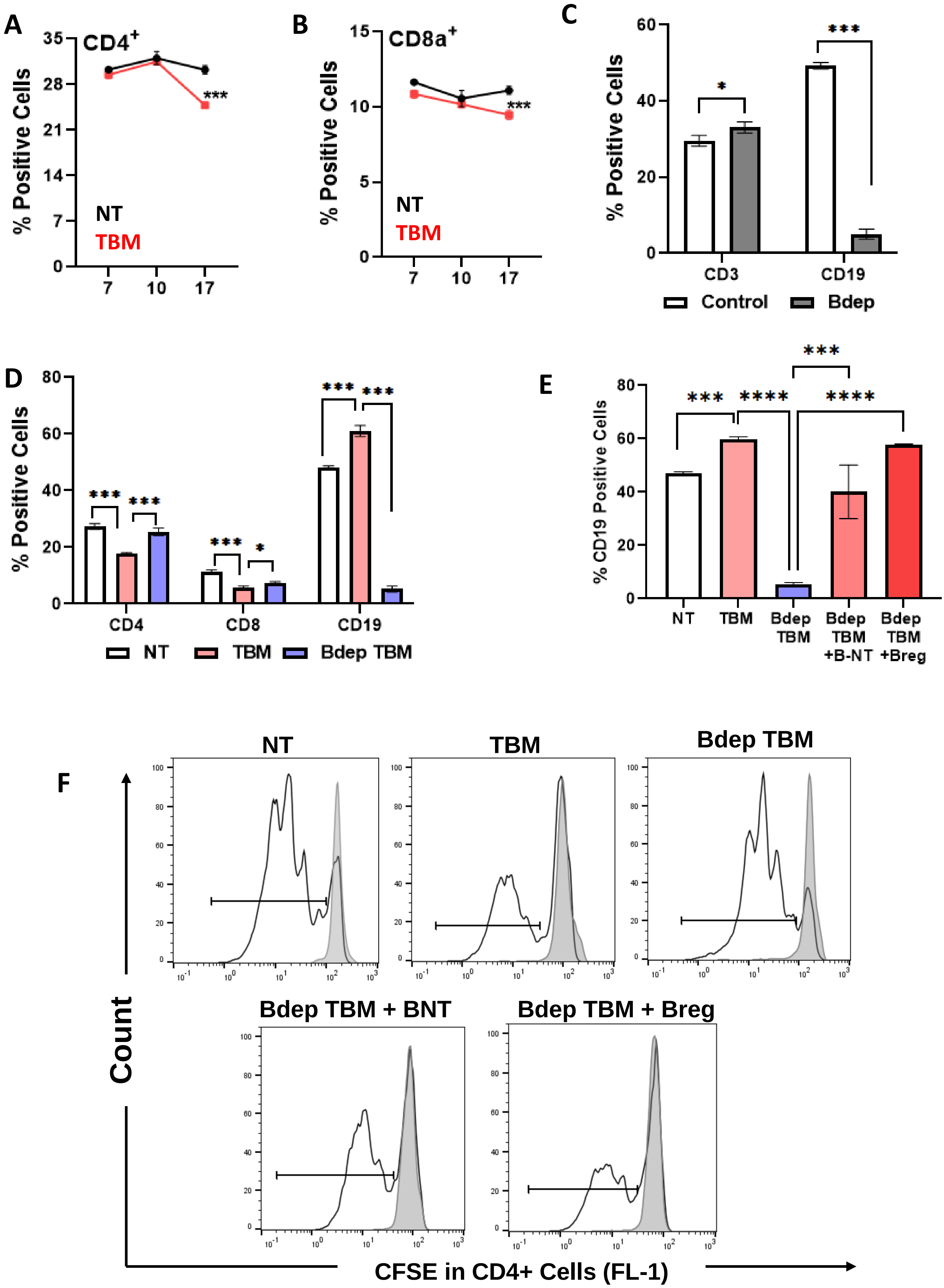

P**Figure S2: B cell depletion in WEHI-164 tumor bearing mice**. Percentages of (A) CD4⁺ and (B) CD8⁺ T cells in TBM on different days after tumor challenge. B cells were depleted in BALB/c mice using anti-CD19 antibody before tumor challenge.(C) Percentages of CD3⁺ T cells and CD19⁺ B cells in Normal and B depleted mice on day before tumor challenge. (D) Percentages of CD4⁺ and CD8⁺ T cells and CD19⁺ B cells on day 21 following tumor challenge. B cells from normal (B-NT) or tumor bearing mice (B-TBM) were adoptively transferred to B depleted tumor bearing mice. (E) Percentage of CD19⁺ B cells in different treatment groups of adoptive transfer experiment. (F) Representative histograms of CFSE dye dilution in T cells from different treatment groups of adoptive transfer experiment.

Figure S3: PGE2-EP2/EP4 signaling induce tBreg mediated suppression

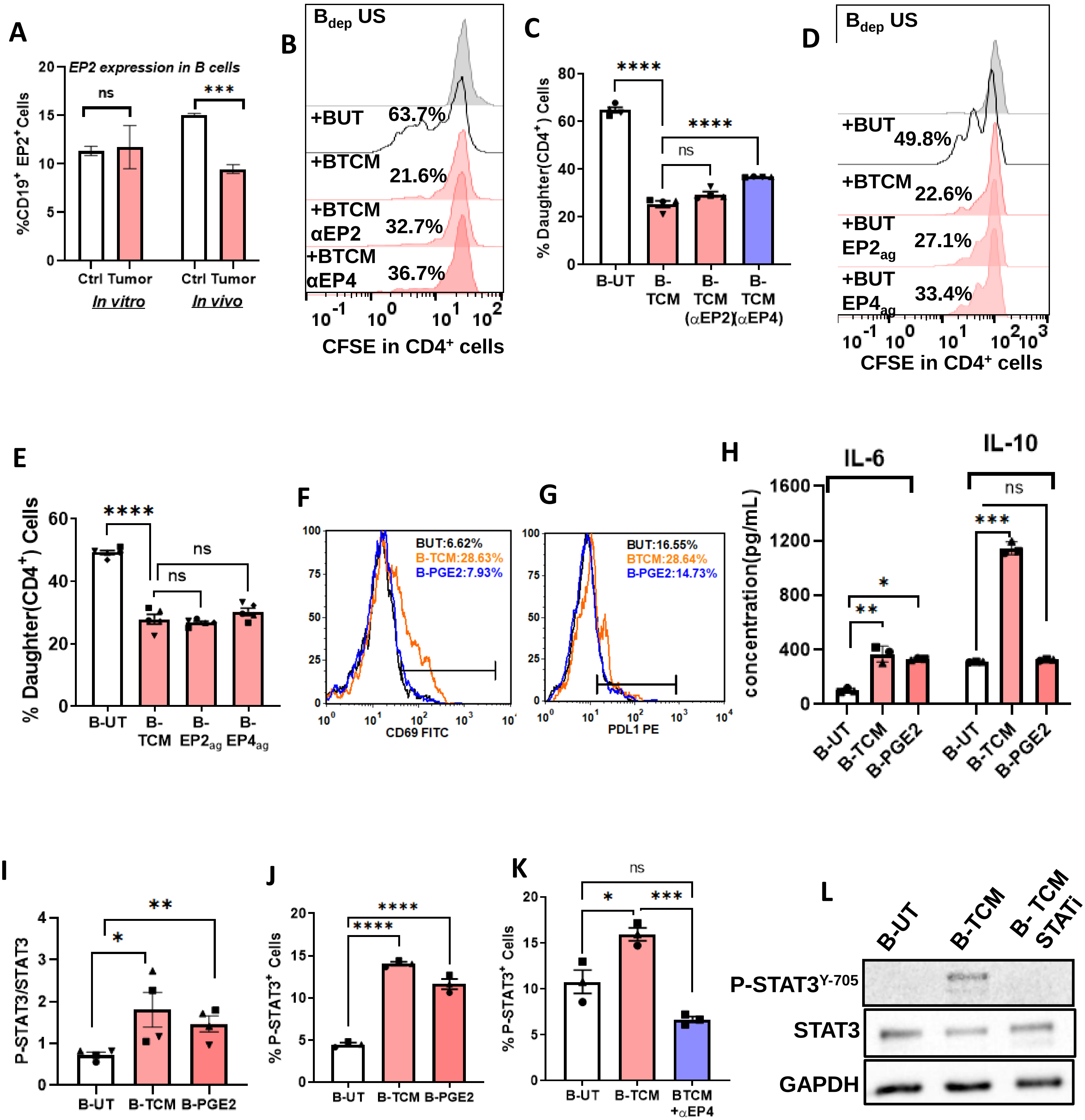

Figure S3: PGE2-EP2/EP4 signaling induce tBreg mediated suppression. (A) Expression of EP2 in B cells in the presence of tumor *in vitro* and *in vivo*. (B-E) T cell responses with tBregs and EP receptor modulation. B cells were cultured with 20% TCM (B-TCM) along with α-EP2 and α-EP4 antibodies or with EP2 and EP4 agonists for 72 h and then co-cultured with CFSE labelled, stimulated Bdep cells. (B) representative flow histograms and (C) bar diagram of $CD4^+$ T cell proliferation with α-EP2 and α-EP4 antibodies (n=4). (D) representative flow histograms and (E) bar diagram of $CD4^+$ T cell proliferation with EP2 and EP4 agonists (n=5). (F-L) Characterization of B-TCM and B-PGE2 cells. Percentages of (F) CD69 and (G) PD-L1 in B-TCM and B-PGE2 cells. (H) IL-6 and IL-10 secretion by B-TCM and B-PGE2 cells (n=3). (I-L) role of STAT3 signaling in tBregs. (I) quantitation of phospho-STAT3 in B-TCM and B-PGE2 cells by western blot (n=4). Percentage of P-STAT3 in (J) B-TCM and B-PGE2 cells and (K) B-TCM and B-TCM+ αEP4 cells analyzed by flow cytometry (n=3). (L) western blot of P-STAT3 with STAT3 inhibitor BP-1-102.

Figure S4: PGE2 induces STAT-3 phosphorylation in Breg via non-canonical MAPK signalling

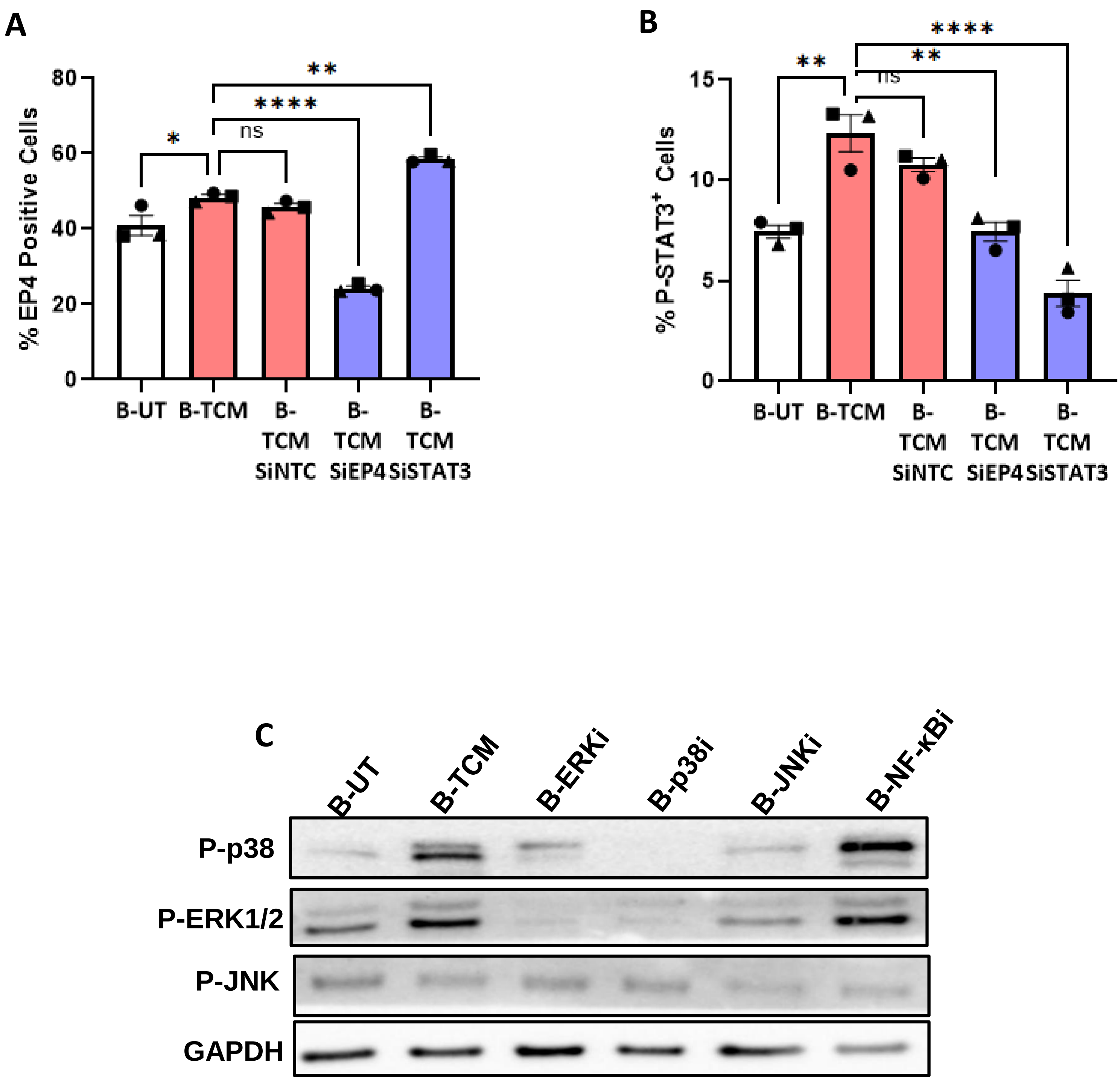

Figure S4: PGE2 induces STAT-3 phosphorylation in Breg via non-canonical MAPK signalling. Purified B cells were transfected with SiNTC, SiEP4 or SiSTAT3 for 24 h followed by culture with 20% TCM. Percentages of (A) EP4 and (B) P-STAT3 in B cells (n=3) . B cells were pre-treated inhibitors of ERK1/2, p38, SAPK/JNK and NF-κB followed by culture with 20% TCM for 72 h. (C) Inhibition of respective proteins evaluated by western blot.

Figure S5: COX-2 inhibitor NS398 averts tumor induced Breg phenotype

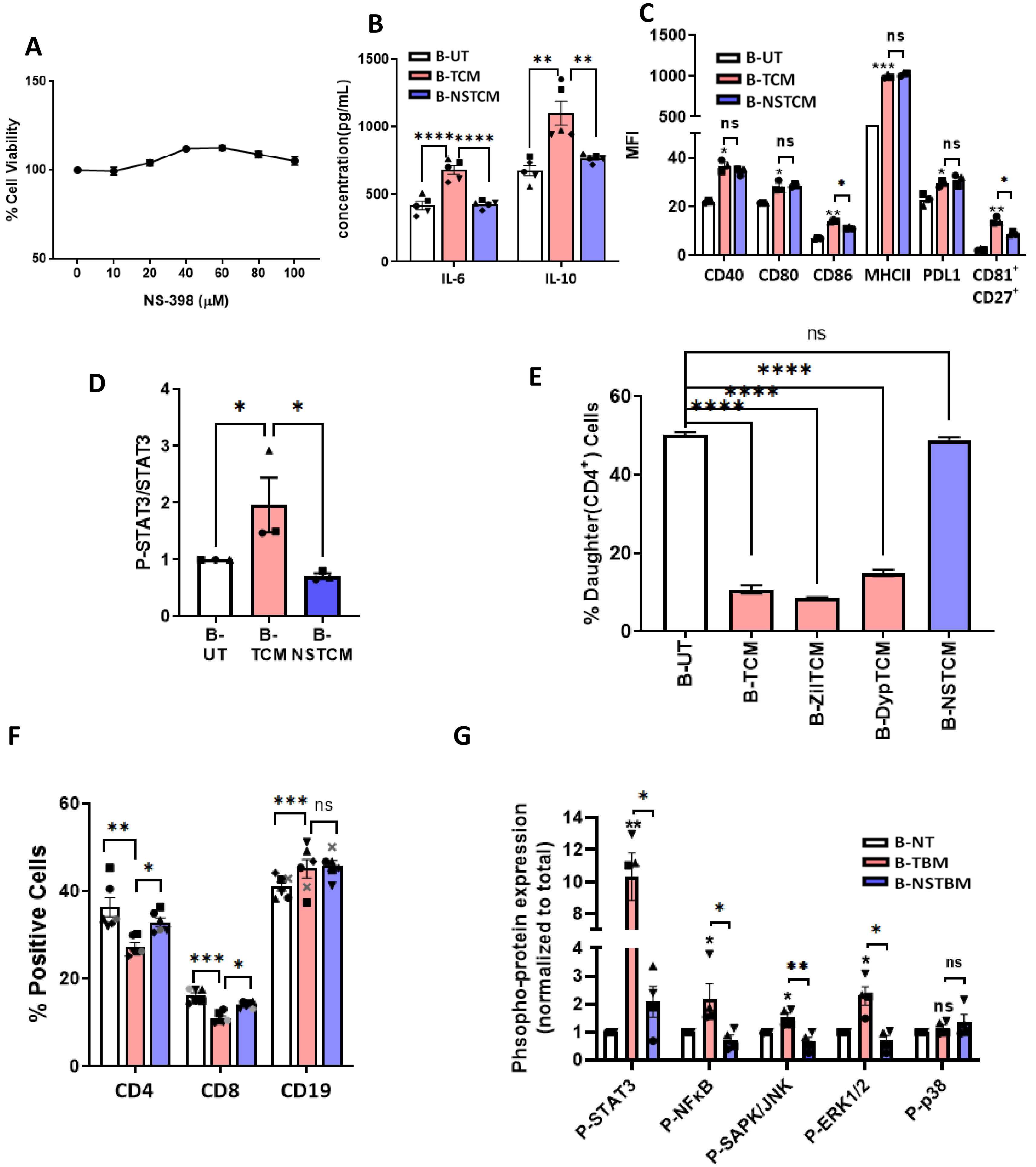

Figure S5: COX-2 inhibitor NS398 averts tumor induced Breg phenotype. (A) Effect of NS-398 on the cell viability of WEHI-164 cells assessed by MTT assay. B cells were cultured with 20% TCM or NS-TCM. (B) Secretion of IL-6 and IL-10 by B-TCM and B-NSTCM cells (n=5). (C) Breg markers were assessed in B-TCM and B-NSTCM by flow cytometry. bar diagram shows MFI of markers (n=3). (D) quantitation of phospho-STAT3 in B-TCM and B-NSTCM cells by western blot (n=3). TCM were generated in presence of inhibitors of arachidonic acid metabolic pathway and used for generation of tBreg. (E) bar diagram showing CD4⁺ T cell proliferation cultured with these B cells (n=3). NS-398 (10mg/Kg body weight) was administered in WEHI-164 TBM for 10 days from day 3 of tumor inoculation. On day 20, the mice were sacrificed for further experiments. (F) Percentages of $CD4^+$, $CD8^+$ and $CD19^+$ cells in spleens of TBM and NS-TBM (n=6). (G) quantitation of signaling proteins in B cells from TBM and NS-TBM by western blot (n=3).

Figure S6: No additional improvement in tumor burden when NS-398 treatment was given in B depleted mice

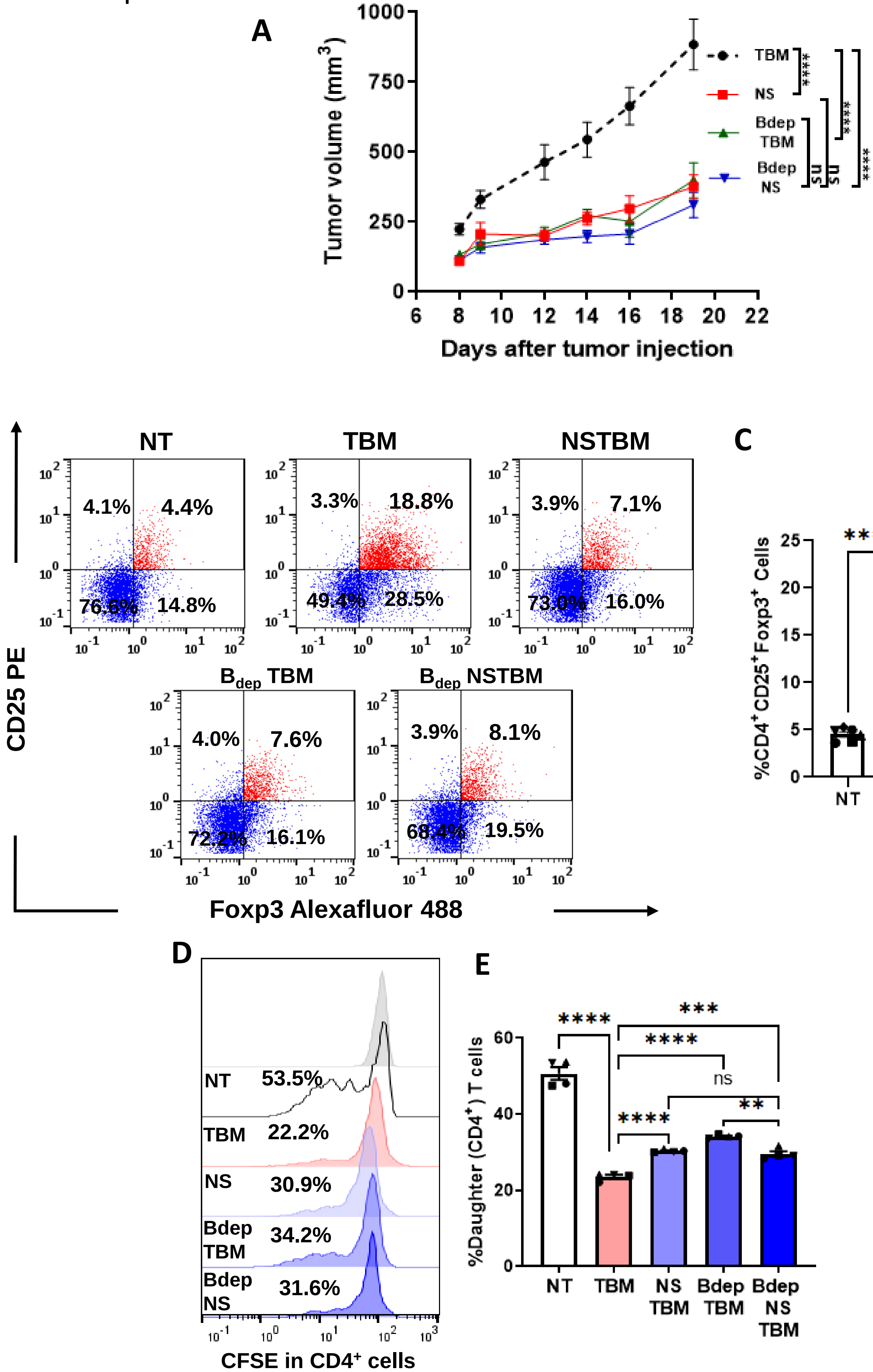

Figure S6: No additional improvement in tumor burden with NS-398 treatment of B depleted tumor bearing mice. B cells were depleted in mice using anti-CD19 antibody three days prior to WEHI-164 tumor inoculation. NS-398 (10mg/Kg body weight) was administered in TBM and Bdep TBM for 10 days from day 3 of tumor inoculation. On day 20, the mice were sacrificed for further experiments. (A) tumor volume measurement. (B) representative dot plots and (C) bar diagram of Treg cells from spleens of treatment groups (n=6). (D) representative flow histogram and (E) bar diagram of CD4⁺ gated T cell proliferation from spleens of treatment groups (n=4). Tumor generation experiments were repeated at least thrice (n=4-6 at each repeat).

Figure S7: TGFB1-Bregs are present in PGE2 high cancers

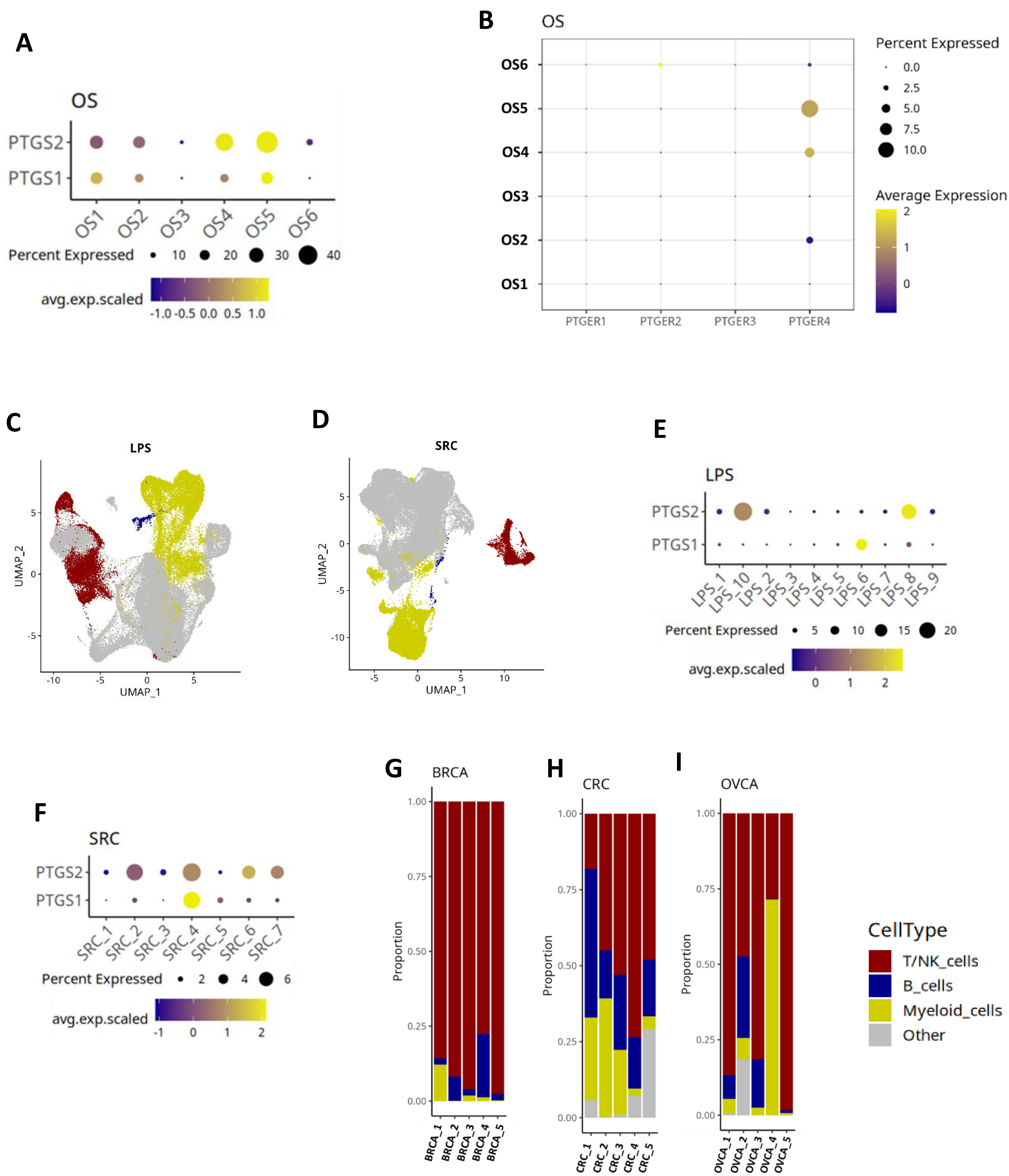

Figure S7: TGF-β expressing Breg cells in PTGS2 high cancers. (A) Bubble plot showing expression of PTGS1 and PTGS2 in individual OS samples. (B) EP receptors PTGER1-4 expression in B cells filtered from individual OS samples. Immune cells (T/NK, B and myeloid cells) were evaluated in cell clusters of human cancers (LPS, SRC, BRCA, CRC and OVCA) using canonical markers. Immune cell proportions in cell clusters of (C) LPS and (D) SRC. Bubbleplot showing PTGS1 and PTGS2 expression in individual samples of (E) LPS and (F) SRC. Bar graphs showing T/NK, B and myeloid cells proportion in (G) BRCA, (H) CRC and (I) OVCA samples.

Figure S8: Breg signature in different cancer type (TCGA)

A

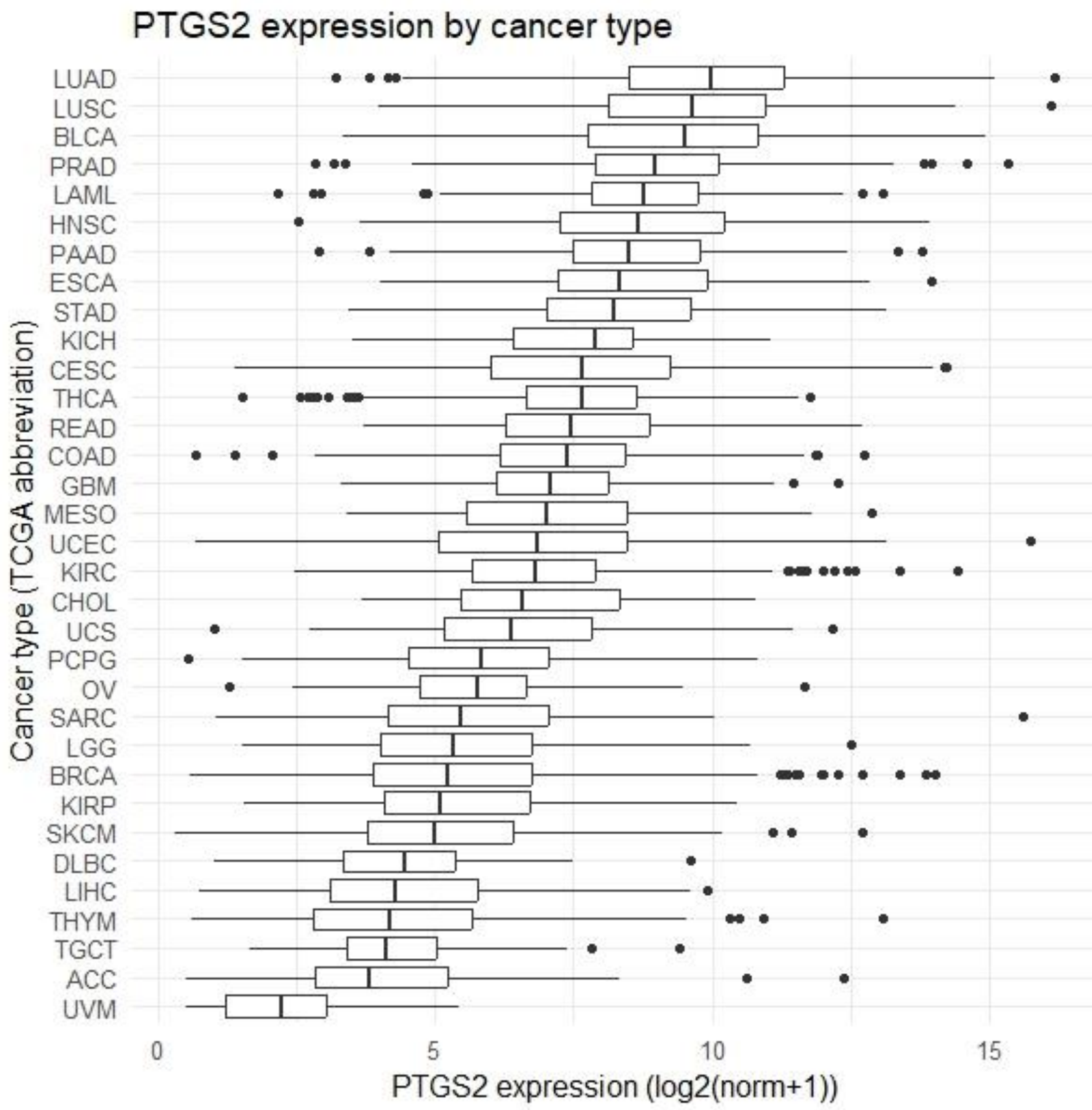

B

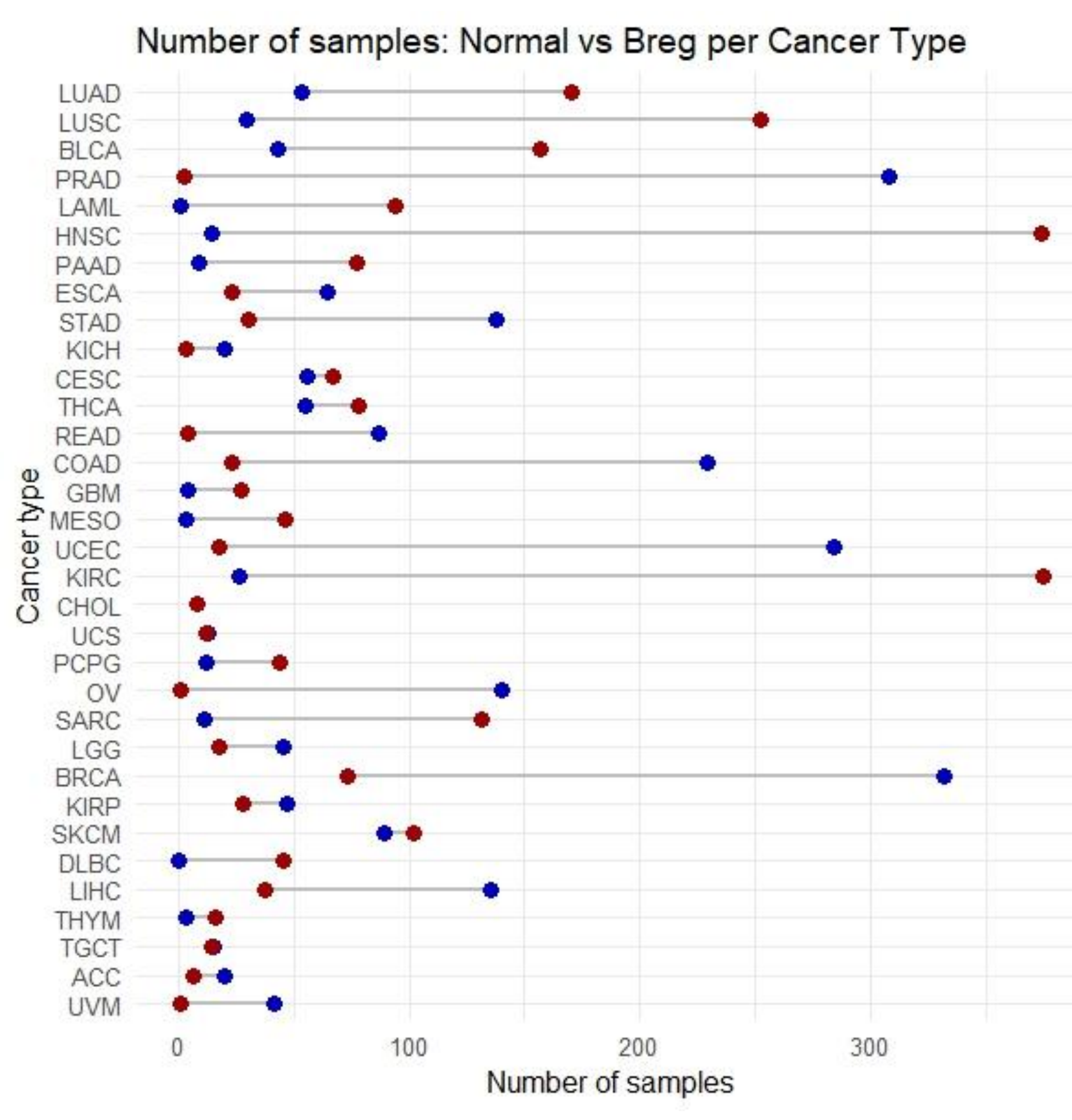

(**Figure S8: Breg signature in different cancer type (TCGA pan-cancer).** A) range of PTGS2 expression in different cancer types. Breg signature was defined as strong B cell marker (CD79A) expression along with high TGFB1 expression(>Q3), where as Normal signature as strong B cell marker (CD79A) expression along with low TGFB1 expression(<Q1). (B) Number of patients showing Normal (blue) and Breg (red) signature in different cancer types.

Figure S9: Granzyme B expression and immune exhaustion in presence of tumor

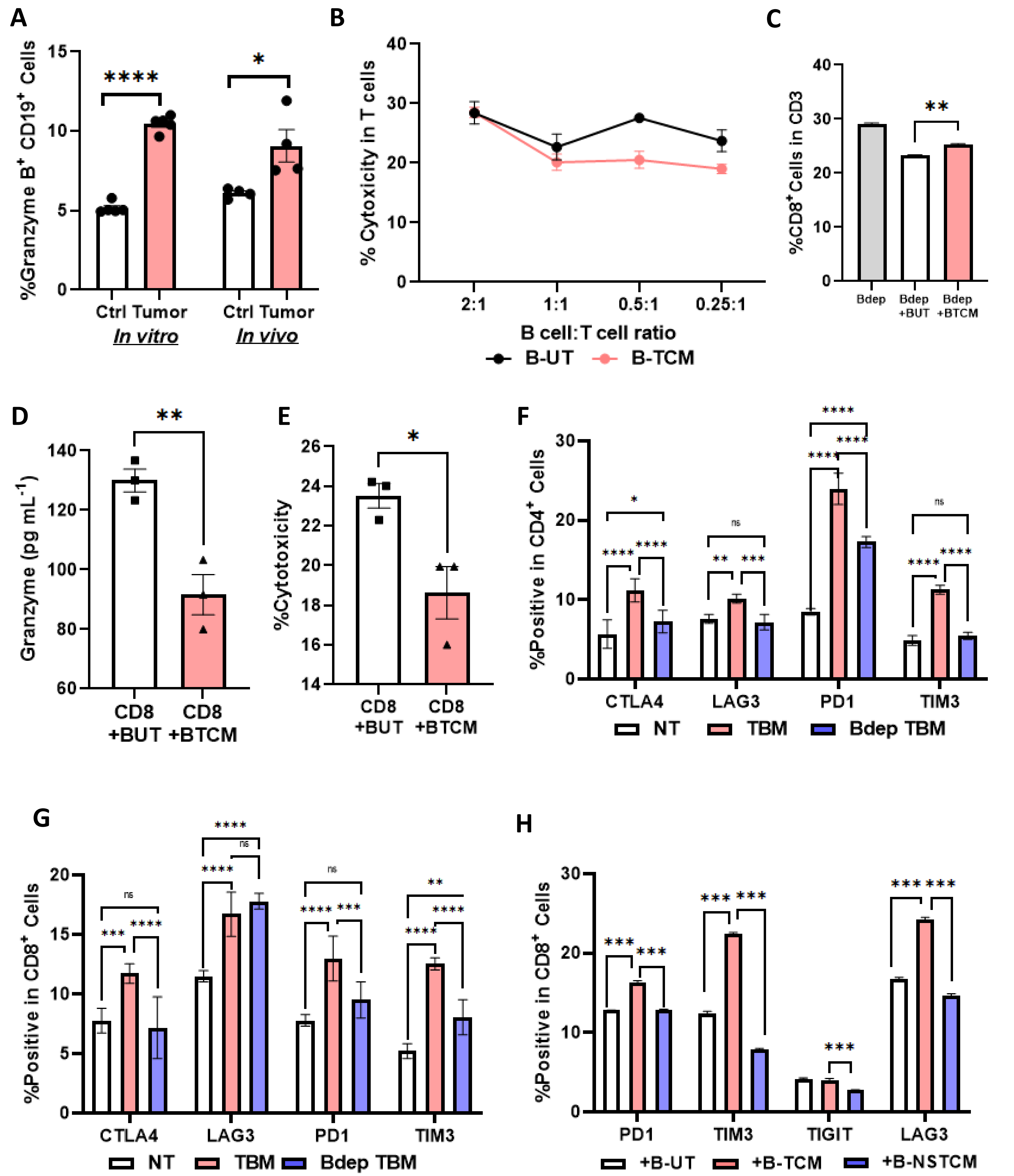

Figure S9: Granzyme expression in B cells and T cell exhaustion in WEHI-164 tumor. (A-E) Effect of Granzyme-B high Breg cells on T cells. (A) Expression of Granzyme-B in B cells treated with TCM or isolated from TBM (n=4). (B) B-UT and B-TCM cells were co-cultured with calcein stained T cells and cytotoxicity was assessed. (n=4). (C) Percentage CD8 cells in B:T co-culture (n=3). T cells were purified from B:T co-culture and incubated with calcein labeled WEHI-164 cells. (D) Granzyme –B expression in T cells, (E) % Cytotoxicity of T cells. (F-H) T cell exhaustion in presence of tumor. Expression of immunosuppressive markers on (F) CD4⁺ and (G) CD8⁺ T cells from the spleens of NT, TBM and Bdep TBM (n=4). bar diagram shows % positive cells (n=3). (H) Purified Bdep T cells were co-cultured with B-UT, B-TCM and B-NSTCM cells for 48 h and expression of immunosuppressive markers were assessed (n=3).

Figure S10: Gating strategy for CFSE dye dilution assay

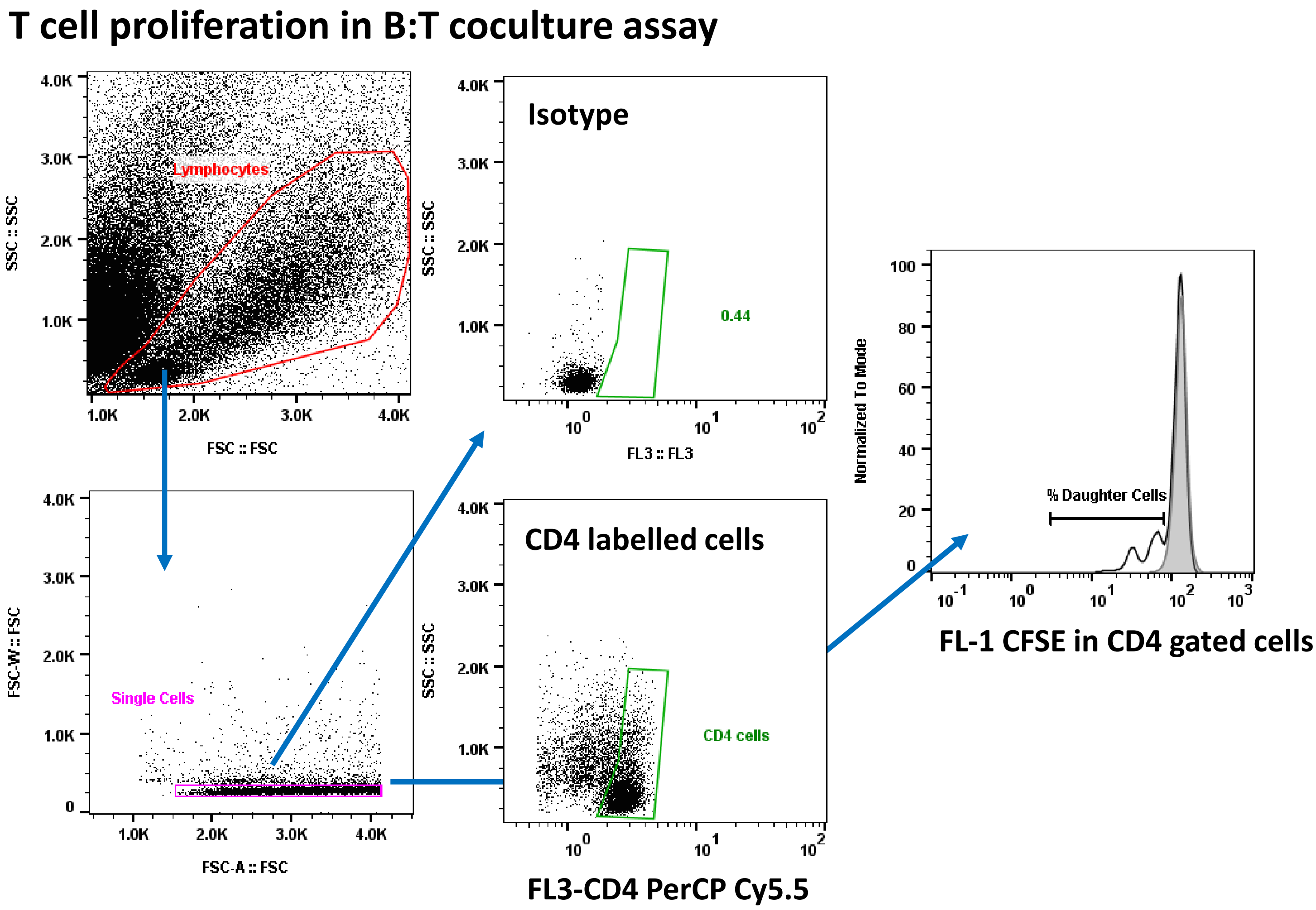

Figure S11: Gating strategy for $CD4^+CD25^+Foxp3^+$ Treg cells

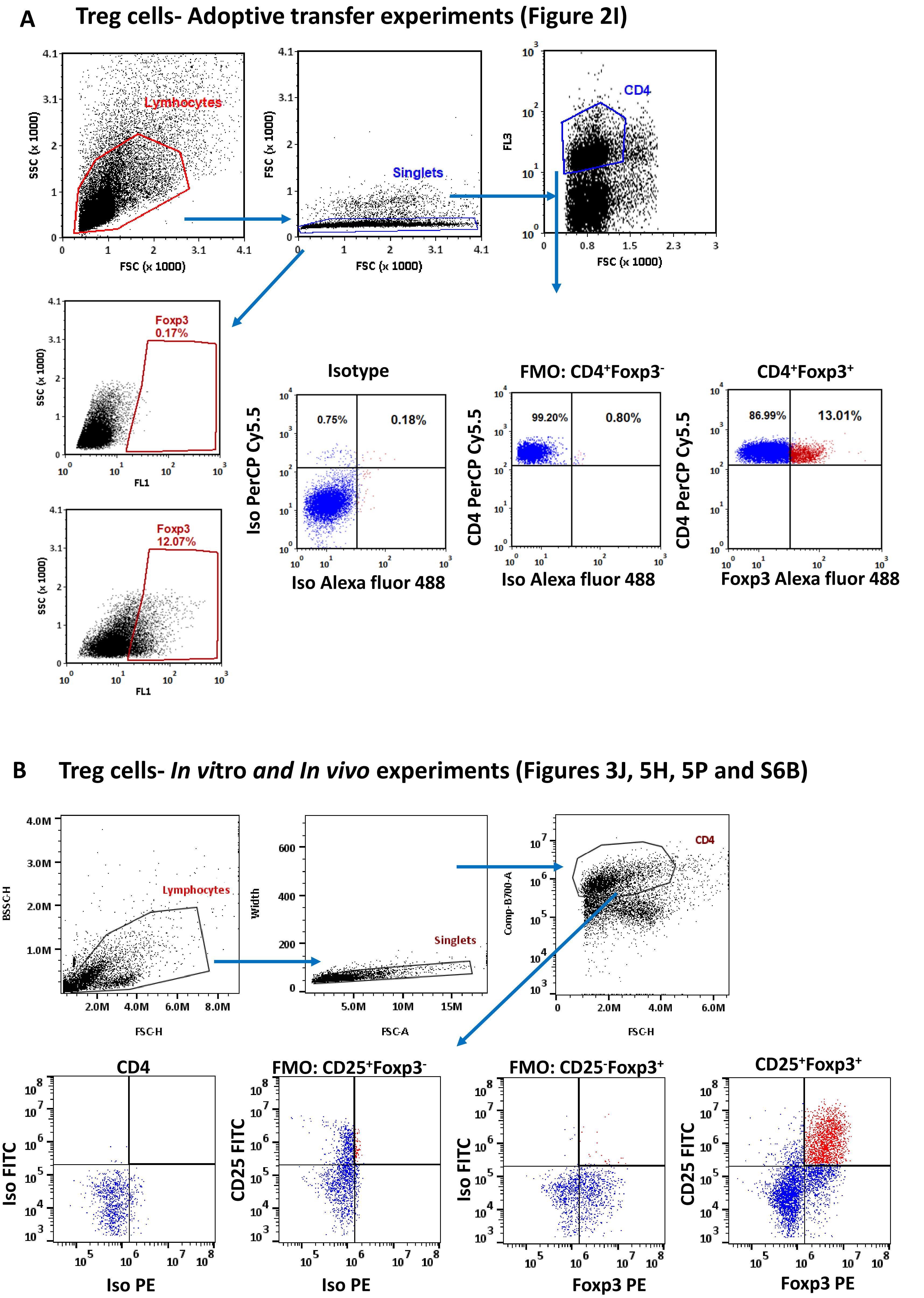

Supplement: Supplementary file 2 [file 41419_2026_9045_MOESM2_ESM.pdf]
